# DNM2 lipid binding drives centronuclear myopathy and represents a potential therapeutic target

**DOI:** 10.1172/jci.insight.204423

**Published:** 2026-05-08

**Authors:** Raquel Gómez-Oca, Xènia Massana-Muñoz, David Reiss, Juliana De Carvalho Neves, Nadege Diedhiou, Roberto Silva-Rojas, Belinda S. Cowling, Marie Goret, Jocelyn Laporte

**Affiliations:** Institute of Genetics and Molecular and Cellular Biology (IGBMC), INSERM U1258, CNRS UMR7104, University of Strasbourg, Illkirch, France.

**Keywords:** Genetics, Muscle biology, Molecular biology, Mouse models, Skeletal muscle

## Abstract

Centronuclear myopathies (CNMs) are rare congenital disorders characterized by muscle weakness, fiber hypotrophy, and organelle mislocalization. Most cases arise from mutations in *MTM1* or *DNM2*, encoding myotubularin and dynamin-2, respectively. DNM2 is a GTPase that binds lipids, oligomerizes around membranes, and mediates fission. We previously showed that DNM2 levels are elevated in *MTM1*-CNM patients and *Mtm1^–/y^* mice, and that normalizing DNM2 rescues disease phenotypes. However, the specific DNM2 functions driving pathology remain unclear. Here, we expressed AAV-delivered WT and DNM2 mutants in WT and *Mtm1^–/y^* mouse muscles to disrupt specific DNM2 molecular functions. In WT mice, overexpression of WT DNM2 and most mutants induced CNM-like phenotypes, including reduced force, fiber hypotrophy, and centralized nuclei, consistent with gain-of-function mechanisms. The lipid-binding-defective mutant K562E did not induce disease-like phenotype. In *Mtm1^–/y^* mice, K562E mutant markedly improved muscle force, mass, and fiber size, while others failed to rescue. Therefore, we generated *Mtm1^–/y^ Dnm2^K562E/+^* mice, which showed full rescue of survival, motor function, and muscle force, with improved muscle mass, fiber size, and organelle positioning despite persistently elevated DNM2 levels. This study reveals that DNM2 lipid binding, not protein abundance or GTPase activity, drives pathology, and represents the most rational therapeutic target for DNM2 therapy in *MTM1*-CNM.

## Abstract 2

In vivo dissection of DNM2 functions identifies lipid binding as the principal driver of myotubular myopathy, revealing a precise therapeutic target.

## Introduction

The dynamin protein family of large GTPases regulates a wide range of essential cellular processes, including membrane trafficking, cytoskeletal remodeling, mitochondrial dynamics, and cytokinesis (1). Mutations in several members of this family cause human diseases that affect distinct tissues (2). The classical dynamins include dynamin-1, which is predominantly expressed in the brain; dynamin-3, expressed in the brain and testis; and dynamin-2 (DNM2), which is ubiquitously expressed (3). These mechanoenzymes share a conserved modular architecture consisting of 4 key domains. The GTPase domain functions as a regulatory switch, cycling between GTP- and GDP-bound states. The stalk domain, composed of the middle and GTPase effector regions, mediates oligomerization and provides a structural link between membranes and the GTPase domain. The pleckstrin homology (PH) domain binds membrane phosphoinositides, while the C-terminal proline-rich domain (PRD) interacts with several SH3-containing proteins to localize and regulate dynamins at specific cellular locations (4).

Biochemical and biophysical studies have revealed a sequential mechanism of dynamin-mediated membrane fission. Dynamin dimerizes via the middle domain, followed by tetramerization. Binding of the PH domain to membranes relieves autoinhibition between the PH and middle domains, permitting higher-order oligomerization into helices around membrane tubules. GTP loading promotes dimerization of the GTPase domains across adjacent helical turns. Subsequent GTP hydrolysis induces conformational changes that constrict the helix and drive membrane fission (5–7). Both lipid and GTP binding enhances dynamin oligomerization and GTPase activity.

Mutational analyses of individual domains have been instrumental in dissecting this mechanism (Supplemental Figure 1A; supplemental material available online with this article; https://doi.org/10.1172/jci.insight.204423DS1). In the GTPase domain, K44A is a classical dominant-negative mutation that strongly reduces GTP affinity, partially inhibiting GTPase and fission activity (8). Structurally, K44A forms more constricted helicoidal assemblies compared with WT (9), and when expressed in cells, it blocks clathrin-mediated endocytosis, lipid droplet breakdown, lamellipodia formation, and actin cytoskeleton remodeling (10–13). Similarly, although K142A retains near-normal GTP hydrolysis, it inhibits the subsequent conformational change, leading to a blockage of endocytic pathways (14). In the middle domain, R399A prevents high-order oligomerization, remaining mostly dimeric. This results in approximately 50% reduced lipid binding and a 90% decrease in GTPase activity, likely due to impaired propagation of conformational changes across oligomers (15). Interestingly, R399A can normalize hyperactive GTPase activity when combined with other pathogenic *DNM2* mutations (16). R465W, another middle domain mutation, conversely promotes excessive oligomerization and GTPase activity (17, 18). In the PH domain, K562E reduces lipid binding and abrogates lipid-induced GTPase activity (19). Deletion of the PRD (ΔPRD) prevents binding to SH3 domain–containing proteins such as amphiphysin-2 (BIN1) or cortactin, altering DNM2 localization and function (20). Although ΔPRD forms helicoidal structures around membrane tubules similarly to WT DNM2 (21), it fails to rescue mitochondrial fission in *DNM2-*knockdown cells (22) or clathrin-mediated endocytosis (23) in dynamin-triple-knockout cells.

Dominant heterozygous mutations in *DNM2* may cause two distinct tissue-specific disorders: centronuclear myopathy (CNM) and Charcot-Marie-Tooth neuropathy (CMT) (24–27). CNM is characterized by muscle weakness and atrophy, muscle fiber hypotrophy, abnormally centralized nuclei, altered oxidative activity linked to mitochondria misposition, sarcomere disorganization, and abnormal triad structure (28, 29). In contrast, CMT presents with motor and sensory neuropathy, characterized by progressive muscle weakness and atrophy, axonal degeneration, and myelin defects (30). The most common CNM mutation, R465W, behaves as a gain-of-function mutant, enhancing oligomerization and GTPase activity. By contrast, the CMT-associated K562E mutation results in decreased lipid binding and GTPase activity (16).

DNM2 overexpression in WT mouse muscle induces a CNM-like phenotype (31). Moreover, high DNM2 protein levels were also found in muscle lysates from patients with X-linked CNM (XLCNM; also called myotubular myopathy; OMIM 310400). XLCNM is the most severe and common form of CNM, caused by loss-of-function mutations in the lipid phosphatase myotubularin (MTM1) (32). Subsequent studies showed that decreasing the expression of DNM2 in both the *Mtm1*-knockout mouse (*Mtm1^–/y^*) and the *Dnm2* R465W knockin mouse (*Dnm2^R465W/+^*) rescued their motor and histopathological phenotypes (32–35).

**Figure 1:**
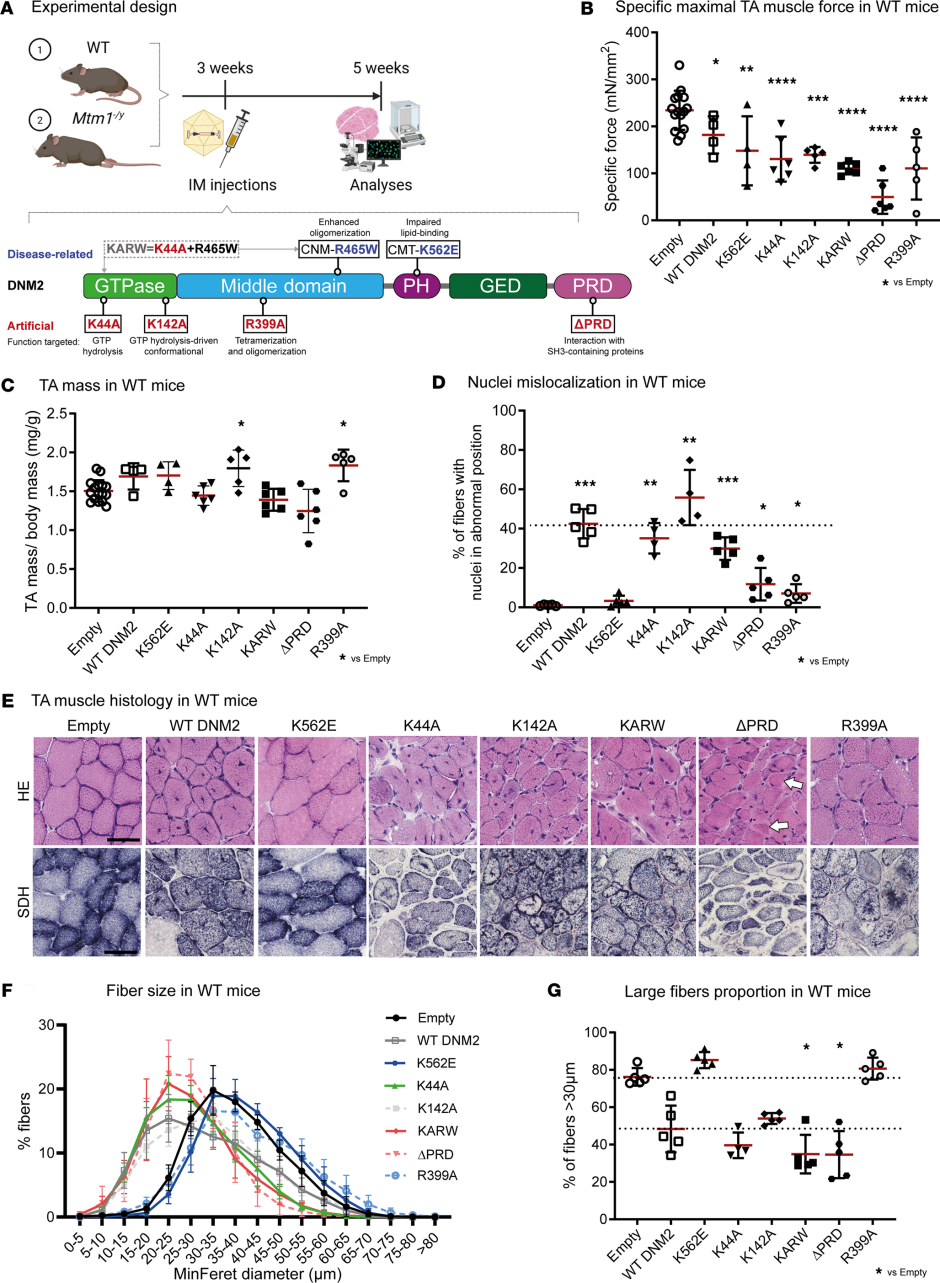
Expression of DNM2 variants in WT muscle induces weakness and differentially recapitulates CNM histopathology. (**A**) Experimental design. Intramuscular AAV delivery of distinct DNM2 variants targeting specific DNM2 domains to disrupt a single DNM2 function. DNM2 R465W and K562E are found in CNM and CMT patients, respectively; KARW K44A, K142A, R399A, and ΔPRD are proof-of-concept mutations. KARW is a combination of the CNM mutation R465W (gain-of-function) with K44A expected to cause GTPase deficiency (loss-of-function). IM, intramuscular; PH, pleckstrin homology; GED, GTPase effector domain; PRD, proline-rich domain. Created in BioRender (Goret M, 2026, https://BioRender.com/lbiksfm). (**B**) Maximal specific force developed by tibialis anterior (TA) muscles at 150 Hz 2 weeks after AAV-DNM2 injection upon stimulation of the sciatic nerve (4 ≤ *n* ≤ 15). (**C**) TA mass normalized to body mass at 5 weeks (4 ≤ *n* ≤ 16). (**D**) Percentage of myofibers with mislocalized nuclei (4 ≤ *n* ≤ 5). (**E**) Transversal sections of TA muscles stained with hematoxylin and eosin (H&E) or succinate dehydrogenase (SDH). Arrows indicate necklace fibers. Scale bars: 50 μm. (**F**) TA fiber size distribution (minimum Feret diameter [MinFeret], 4 ≤ *n* ≤ 5). (**G**) Proportion of large fibers (MinFeret > 30 μm) in transversal TA sections (4 ≤ *n* ≤ 5). Each dot represents a mouse. Values are represented as mean ± SD; *P < 0.05, **P < 0.01, ***P < 0.001, ****P < 0.0001. (**B**) Ordinary 1-way ANOVA. (**D**) Brown-Forsythe ANOVA. (**C** and **G**) Kruskal-Wallis test.

While increased DNM2 levels and activity have been implicated in CNM pathogenesis, it has not been resolved whether lipid binding, GTPase activity, oligomerization, or protein abundance represents the principal pathogenic driver in vivo. In this study, we systematically disrupted distinct DNM2 functional domains to determine which activity drives CNM pathology and thus represents the most relevant target for modulation of the CNM pathology linked to MTM1 defect. As proof of concept, we injected adeno-associated viruses (AAVs) carrying DNM2 variants (WT, K44A, K142A, R399A, R465W, K562E, ΔPRD, and the double mutant K44A+R465W [KARW]; Figure 1) into skeletal muscle of WT and *Mtm1^–/y^* mice. We assessed muscle force and mass and fiber morphology and histology, as well as membrane protein localization and myofiber ultrastructure, and correlated them with molecular investigations. All variants caused weakness in WT muscles and, except for the lipid-binding-deficient K562E mutant, induced CNM-like histopathology. In *Mtm1^–/y^* mice, most variants either failed to rescue disease features or worsened nuclear mislocalization, while K562E mutant improved muscle force, mass, and fiber size. The double-mutant *Mtm1^–/y^ Dnm2^K562E/+^* mice showed full rescue of lifespan, muscle force, histopathology, and ultrastructural integrity, despite persistently elevated DNM2 levels. Our results indicate that CNM is primarily driven by increased DNM2 levels, independently of GTPase activity, with lipid binding driving the histopathology. Disruption of DNM2 GTPase function, oligomerization, or effector interactions fails to mitigate *Mtm1*-CNM, whereas selective impairment of lipid binding fully rescues the phenotype. Our findings establish DNM2 lipid binding as the principal pathogenic activity driving CNM phenotypes in vivo and suggest that selective modulation of this function represents a rational avenue for future therapeutic development.

## Results

### Expression of DNM2 variants in WT muscle induces weakness and differentially recapitulates CNM histopathology.

To identify the contribution of DNM2 activities in healthy muscle and the main molecular dysfunction(s) underlying CNM, we created AAV expressing DNM2 WT or DNM2 with mutations disrupting distinct steps of the dynamin functional cycle. We also combined the K44A and R465W mutants (KARW construct) in an attempt to balance the gain-of-function effect of the R465W CNM mutation by decreasing the GTP binding. The AAV constructs were injected at a dose of 1.5 × 10^9^ viral genomes in tibialis anterior (TA) muscle in 3-week-old WT mice, and analyses were performed 2 weeks after injection (Figure 1A). DNM2 mutations used are described in Figure 1A and Supplemental Figure 1A. We focused on male mice and injection at 3 weeks to be able to compare with subsequent experiments performed in the X-linked *Mtm1*-CNM mice dying by adulthood.

Overexpression of the different DNM2 variants in WT muscles was confirmed by Western blot, showing levels about 5–10 times the level of endogenous DNM2 (Supplemental Figure 1B). The overexpression level was similar among the different constructs. All DNM2 variants caused decrease in the specific maximal force of the injected muscle compared with injection of an empty AAV; the stronger effect was seen with the ΔPRD mutant (Figure 1B). Overexpression of WT DNM2 protein also decreased muscle force. There was no significant muscle atrophy (Figure 1C).

To investigate which DNM2 function is linked to the typical CNM histopathology, TA muscle sections were stained with hematoxylin and eosin (H&E; for general histological aspect) and succinate dehydrogenase (SDH; indicative of the oxidative activity of mitochondria). Overexpression of WT DNM2 induced typical CNM hallmarks with marked centralization and internalization of nuclei, central accumulation of oxidative activity, and a tendency toward smaller fibers, as we previously reported (31, 36) (Figure 1, D–G, Supplemental Figure 1C, and Supplemental Figures 2 and 3). Similar phenotypes were observed in patients and in mouse models with knockin of CNM mutations (37, 38). These data confirm that increased function of DNM2 causes CNM and that CNM mutations have a gain-of-function effect. Unexpectedly, mutations responsible for the inhibition of the GTPase activity (K44A) or GTP-induced conformational change (K142A) still correlated with CNM-like histology, similarly to overexpression of WT DNM2. Moreover, inhibition of the GTPase activity in the context of a gain-of-function CNM mutant (KARW double mutant) did not prevent the establishment of the CNM phenotypes. Deletion of the PRD (ΔPRD) also resulted in a CNM-like histology, with hypotrophic fibers with internal nuclei and necklace fibers (Figure 1E, arrows), i.e., sub-sarcolemmal basophilic rings composed of mitochondria, which are typically observed in patients with late-onset *MTM1*- and *DNM2*-related CNM (39–41). Blocking dynamin at the dimeric state (R399A) did not induce fiber hypotrophy but correlated with a slight increase in mislocalized nuclei and a strong decrease in oxidative activity. Conversely, overexpression of the lipid-binding-defective K562E CMT mutant did not cause histological abnormalities or fiber hypotrophy, confirming previous results (36).

Overall, the CNM phenotype due to DNM2 gain of function, i.e., overexpression of WT-DNM2, is recapitulated despite the lack of GTP-dependent activation of dynamin. However, lipid binding appears to be essential in the pathological process, as the K562E mutant did not induce a CNM-like histology.

### Expression of DNM2 variants in WT muscle impairs myofiber ultrastructure and intracellular protein dynamics.

Dynamins play key roles in membrane trafficking, remodeling, and protein homeostasis (42). We hypothesized that the CNM-like phenotypes induced by overexpression of WT DNM2 or certain mutants result from defects in these cellular processes.

**Figure 2:**
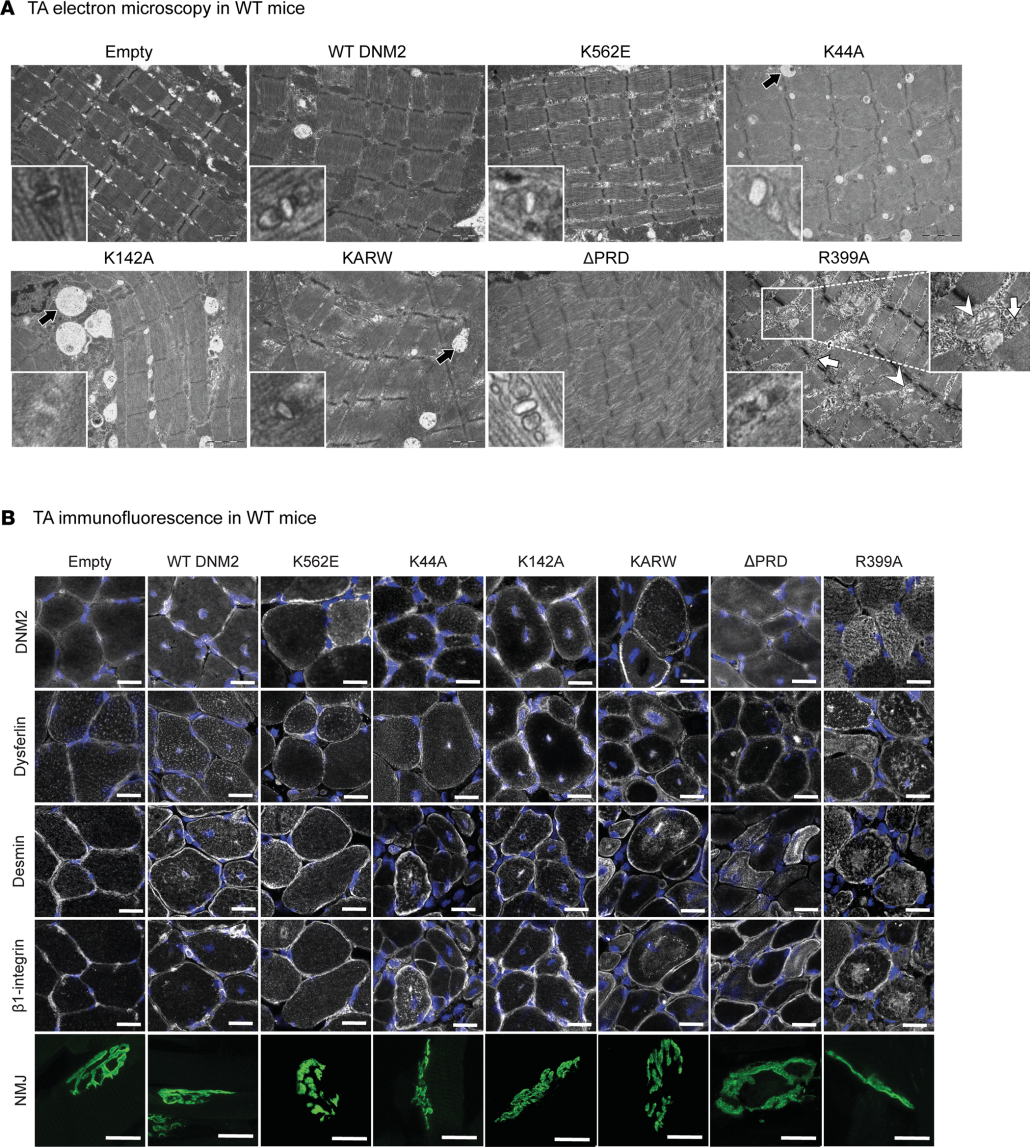
Expression of DNM2 variants in WT muscle impairs myofiber ultrastructure and intracellular protein dynamics. (**A**) Electron microscopy images of longitudinal TA sections, injected with DNM2 constructs, showing overall sarcomere organization. Black arrows indicate enlarged vacuoles, white arrowheads indicate mitochondria with disrupted cristae, and white arrows indicate glycogen accumulation. Scale bars: 2 μm. Zoomed views of triads in bottom-left corners (×8 relative to main image). (**B**) Immunolabeling of DNM2, dysferlin, desmin, and β_1_-integrin in transversal TA sections, and α-bungarotoxin staining of neuromuscular junctions (NMJs) in longitudinal TA sections. Scale bars: 20 μm.

To test this, myofiber organization was examined by electron microscopy. While muscles injected with empty AAV or K562E maintained normal sarcomere organization, expression of WT DNM2 or the other mutants caused disorganized sarcomeres with, in some cases, Z-line misalignment (Figure 2A). Notably, enlarged vacuoles were observed with K44A, K142A, and KARW expression (black arrows). Consistent with histological findings and reduced oxidative activity, the R399A mutant produced a particularly severe phenotype, including abnormal mitochondria with disrupted cristae (white arrowheads) and glycogen accumulation (white arrows). Triads, critical for excitation-contraction coupling, were normally localized on both sides of the Z-line in muscles expressing WT, K562E, R399A, or ΔPRD, although T tubules seemed dilated in comparison with empty controls (Figure 2A, bottom left). In contrast, triads were rare and often malformed in GTPase-related mutants (K44A, K142A, KARW). We conclude that expression of all DNM2 proteins impacted on membrane remodeling at the triad.

To further assess whether distinct DNM2 functions influence membrane trafficking in muscle, we first examined the localization of β_1_-integrin, a key focal adhesion protein that undergoes dynamin-dependent endocytosis (43) and is known to accumulate in both *Mtm1^–/y^* and *Dnm2*-CNM models (44, 45). In muscles injected with DNM2 variants, β_1_-integrin displayed increased cytosolic staining, frequently forming central accumulations or necklace-like patterns. This phenotype was observed across all mutants, although K562E-expressing muscles displayed only small and less frequent accumulations (Figure 2B). We next analyzed dysferlin, a protein involved in membrane remodeling and repair that is mislocalized in CNM (37). Cytosolic accumulations of dysferlin were observed in most conditions, with the exception of K562E. To evaluate whether DNM2 activity impacts protein homeostasis, we assessed desmin, an intermediate filament protein found aggregated in *Mtm1*- and *Dnm2*-CNM mouse models and patients (45–48). Desmin localization was altered with expression of DNM2 WT and all mutants except K562E, with central aggregation in most of the cases, suggesting degradation defects. These immunofluorescence analyses were performed at high magnification to visualize subcellular distribution patterns and therefore provide qualitative assessment of protein mislocalization. We then investigated neuromuscular junctions (NMJs), essential interfaces for transmitting signals from motor neurons to muscle fibers. Using fluorescently labeled α-bungarotoxin, we examined NMJ morphology in transduced muscles (Figure 2B and Supplemental Figure 1, D and E). Muscles expressing WT DNM2 or the K142A mutant displayed a tendency toward elongated (reduced circularity) and fragmented NMJs compared with WT-empty controls. K44A-expressing muscles showed a modest increase in fragmentation, whereas K562E, KARW, ΔPRD, and R399A mutants exhibited only small, non-significant changes in NMJ morphology. Given the limited sampling and inherent variability of NMJ morphology, these observations should be interpreted cautiously. We hypothesize that mislocalization of intracellular organelles and proteins arises both from impaired DNM2 functions and from abnormal DNM2 distribution itself. In AAV-empty and K562E-expressing muscles, DNM2 showed a normal sarcolemmal distribution. In contrast, most other mutants and WT DNM2 also accumulated centrally in addition to the sarcolemmal localization, coinciding with nuclei centralization. The R399A mutant displayed an abnormal diffuse cytosolic distribution consistent with marked intracellular disorganization. Because the injected DNM2 variants were untagged so as not to interfere with their activity, exogenous and endogenous protein cannot be distinguished. Nonetheless, the selective mislocalization observed in mutant-injected muscles indicates involvement of the exogenously expressed proteins, although contribution from endogenous DNM2 cannot be excluded.

These results suggest that overexpression of WT DNM2 and all DNM2 variants, except K562E, disrupts DNM2 protein distribution and impairs membrane trafficking, remodeling, and protein homeostasis, likely affecting organelle positioning, fiber morphology, and ultrastructure and ultimately altering muscle force.

### Overexpression of the lipid-binding-defective DNM2 K562E mutant improves TA muscle force and atrophy in Mtm1^–/y^ mice.

Building on our previous results highlighting the role of DNM2 functions in the CNM pathology, we next asked which specific DNM2 property could be targeted to rescue muscle phenotypes in the *Mtm1^–/y^* mouse, a faithful model of severe X-linked CNM (XLCNM). These mice develop progressive myopathy from 2–3 weeks of age, characterized by reduced muscle force, atrophy, and typical CNM histology, and generally die around 7–8 weeks (49).

We exogenously expressed WT and the previous DNM2 mutants in the TA of 3-week-old *Mtm1^–/y^* mice and assessed muscle function and structure 2 weeks later (Figure 1A). Overexpression of DNM2 proteins was confirmed by Western blot, reaching approximately 5–10 times the endogenous level found in AAV-empty–injected muscles (Supplemental Figure 1F).

**Figure 3:**
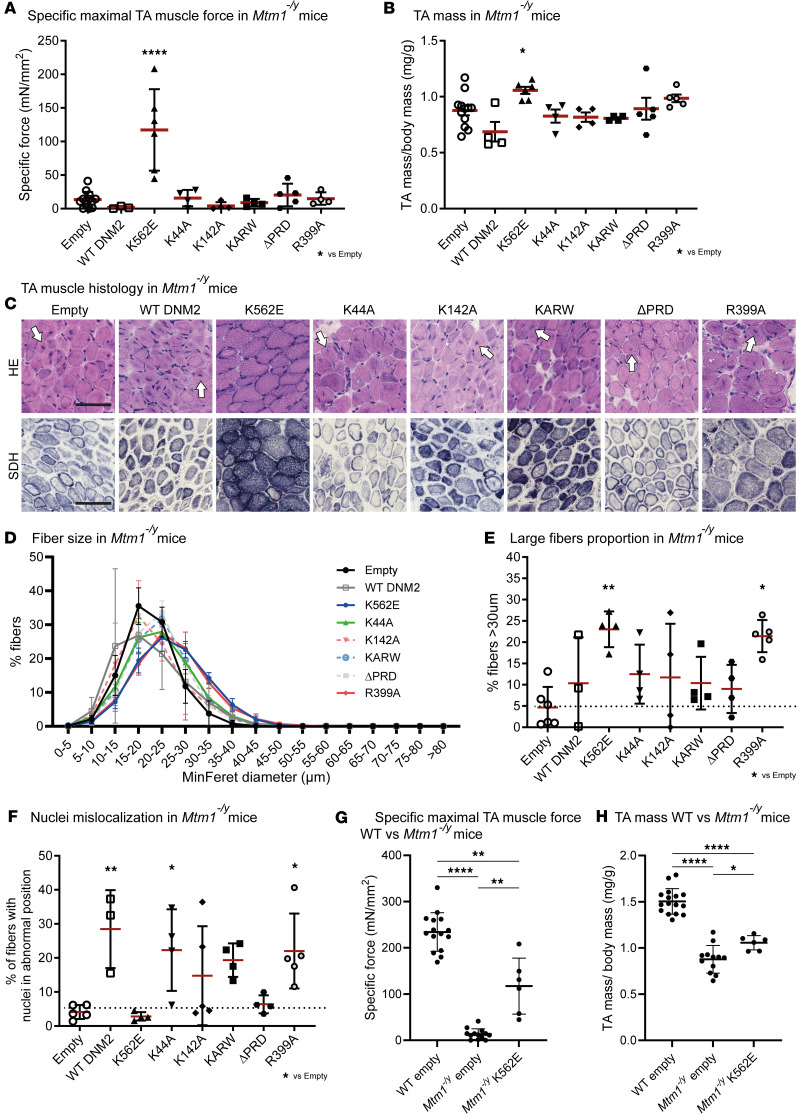
Overexpression of the lipid-binding-defective DNM2 K562E mutant improves TA muscle force and atrophy in *Mtm1^–/y^* mice. (**A**) Maximal specific force developed by *Mtm1^–/y^* TA muscles at 150 Hz 2 weeks after AAV-DNM2 injection upon stimulation of the sciatic nerve (3 ≤ *n* ≤ 13). (**B**) TA mass normalized to body mass at 5 weeks (4 ≤ *n* ≤ 12). (**C**) Transversal sections of *Mtm1^–/y^* TA muscles expressing DNM2 mutants, stained with H&E and SDH. White arrows: necklace fibers. Scale bars: 50 μm. (**D**) Distribution of TA fibers based on their MinFeret diameter (3 ≤ *n* ≤ 6). (**E**) Proportion of large fibers (MinFeret > 30 μm) in *Mtm1^–/y^* transduced TA sections (3 ≤ *n* ≤ 6). (**F**) Percentage of myofibers with mislocalized nuclei (3 ≤ *n* ≤ 5). (**G**) Maximal specific force developed by *Mtm1^–/y^* TA muscles at 150 Hz with or without DNM2 K562E expression compared with WT controls (6 ≤ *n* ≤ 15). (**H**) TA mass normalized to body mass in the same groups (6 ≤ *n* ≤ 16). Each dot represents 1 mouse. Values are shown as mean ± SD; *P < 0.05, **P < 0.01, ****P < 0.0001. (**A**, **F**, and **H**) Ordinary 1-way ANOVA. (**G**) Brown-Forsythe ANOVA. (**B** and **E**) Kruskal-Wallis test.

Expression of WT DNM2 did not improve muscle force nor mass and tended to slightly worsen these phenotypes (Figure 3, A and B). In contrast, the lipid-binding-defective K562E mutant significantly improved both specific muscle force and muscle mass, whereas all other mutants had no effect. To assess whether functional improvements were accompanied by histological and morphological rescue, we performed H&E and SDH staining and quantified fiber size and nuclear positioning (Figure 3C). Expression of K562E markedly increased fiber size and improved histological findings, while the R399A oligomerization mutant enhanced fiber size but worsened nuclear mislocalization (Figure 3, D–F, Supplemental Figure 1G, and Supplemental Figures 4 and 5). Conversely, the ΔPRD did not increase fiber size and did not worsen nucleus mislocalization. The other mutants did not ameliorate the histopathology of *Mtm1^–/y^* mice. Nuclear mislocalization was even exacerbated with WT and K44A injections.

The lipid-binding-defective DNM2 K562E mutant was the only one that improved muscle force, mass, fiber size, and overall histopathological appearance in *Mtm1^–/y^* mice. The rescue was partial after 2 weeks of treatment, as muscle force and mass reached intermediate levels between untreated *Mtm1^–/y^* mice and healthy controls (Figure 3, G and H).

### Overexpression of the lipid-binding-defective DNM2 K562E mutant improves ultrastructure and intracellular protein dynamics in Mtm1^–/y^ mice.

As we previously found that membrane and protein homeostasis are key pathways altered in the CNM disease, we investigated whether improvement of these pathways is the basis of the amelioration in muscle force and histology. In the *Mtm1^–/y^* mouse, myofiber disorganization, abnormal triad shape and localization, desmin aggregation, and β_1_-integrin intracellular retention were previously described (44, 47, 49, 50).

**Figure 4:**
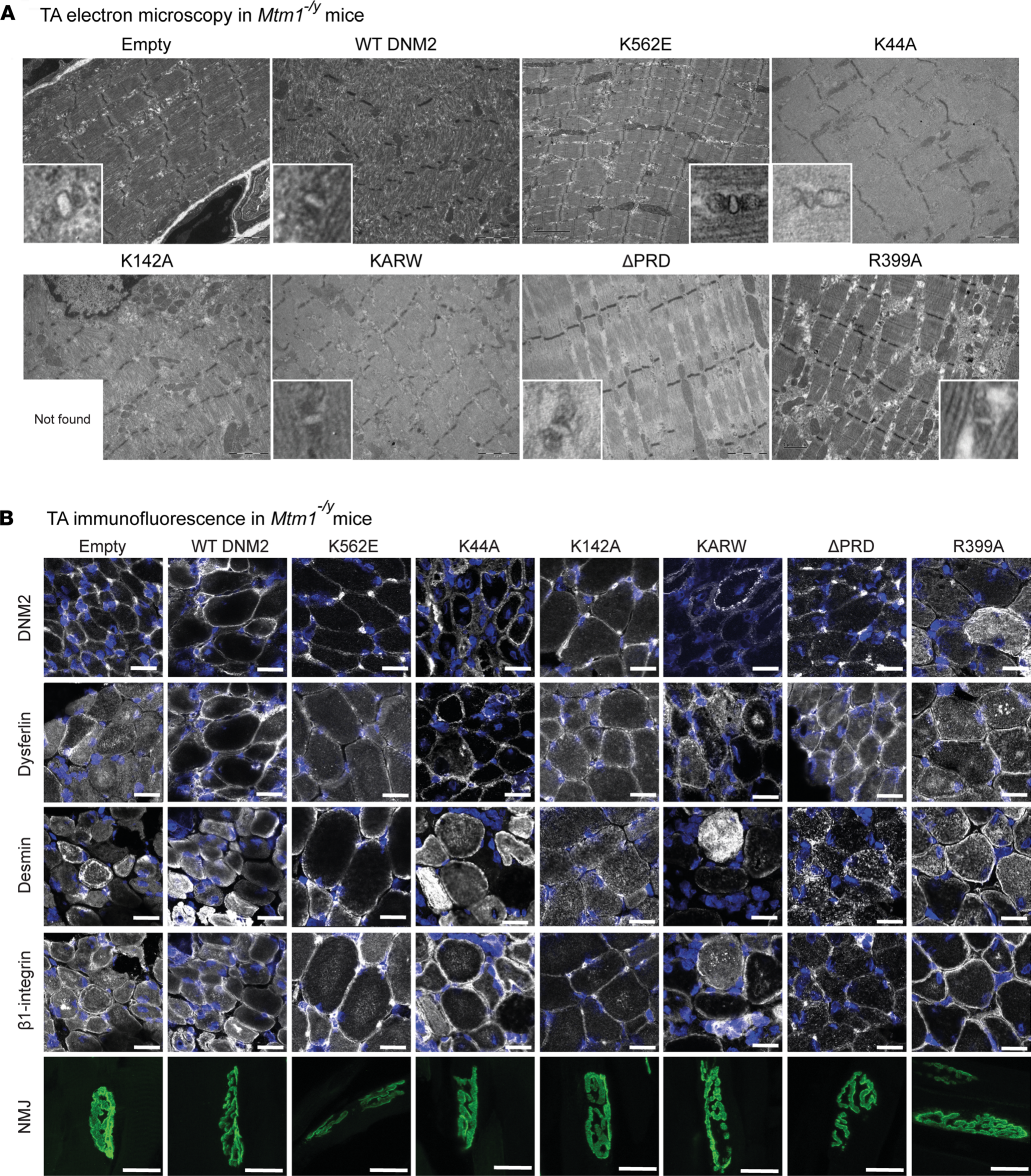
Overexpression of the lipid-binding-defective DNM2 K562E mutant improves ultrastructure and intracellular protein dynamics in *Mtm1^–/y^* mice. (**A**) Electron microscopy images of longitudinal *Mtm1^–/y^* TA sections, injected with DNM2 constructs, showing overall sarcomere organization. Scale bars: 2 μm (1 μm for R399A). Zoomed views of triads in bottom corners (×8 relative to main image). (**B**) Immunolabeling of DNM2, dysferlin, desmin, and β_1_-integrin in transversal TA sections, and α-bungarotoxin staining of NMJs in longitudinal TA sections. Scale bars: 20 μm.

Ultrastructural analysis revealed that K562E and ΔPRD partially restored general myofiber architecture in comparison with AAV-empty controls (Figure 4A). Some residual defects remained: K562E showed occasional dilated T tubules, while ΔPRD exhibited minor Z-line misalignment. All other mutants displayed persistent abnormalities: WT DNM2 caused general disorganization; K44A, K142A, and KARW showed Z-line misalignment; and R399A had enlarged inter-sarcomeric spaces. T tubules were slightly enlarged in all conditions, while no triads were recognized in K142A.

Analysis of intracellular organization and protein localization revealed that desmin, β_1_-integrin, and dysferlin accumulations persisted with most injected mutants, whereas fibers expressing K562E were the least affected, displaying markedly fewer intracellular accumulations than empty controls (Figure 4B).

Taken together, the positive effect of the lipid-binding-defective mutant K562E on *Mtm1^–/y^* muscle force, atrophy, and histopathology correlated with improvement in ultrastructure, membrane trafficking, and protein homeostasis.

Overall, in WT mice, all DNM2 variants except K562E induced muscle weakness and CNM-like phenotypes, whereas in *Mtm1^–/y^* mice, the lipid-binding-defective K562E mutant exerted a beneficial effect, while exogenous expression of the other loss-of-function mutants was ineffective or deleterious (Table 1 and Supplemental Figure 6).

### Systemic expression of the CMT neuropathy mutant K562 restores muscle function and ameliorates structural and histopathological defects in Mtm1^–/y^ Dnm2^K562E/+^ mice.

**Figure 5:**
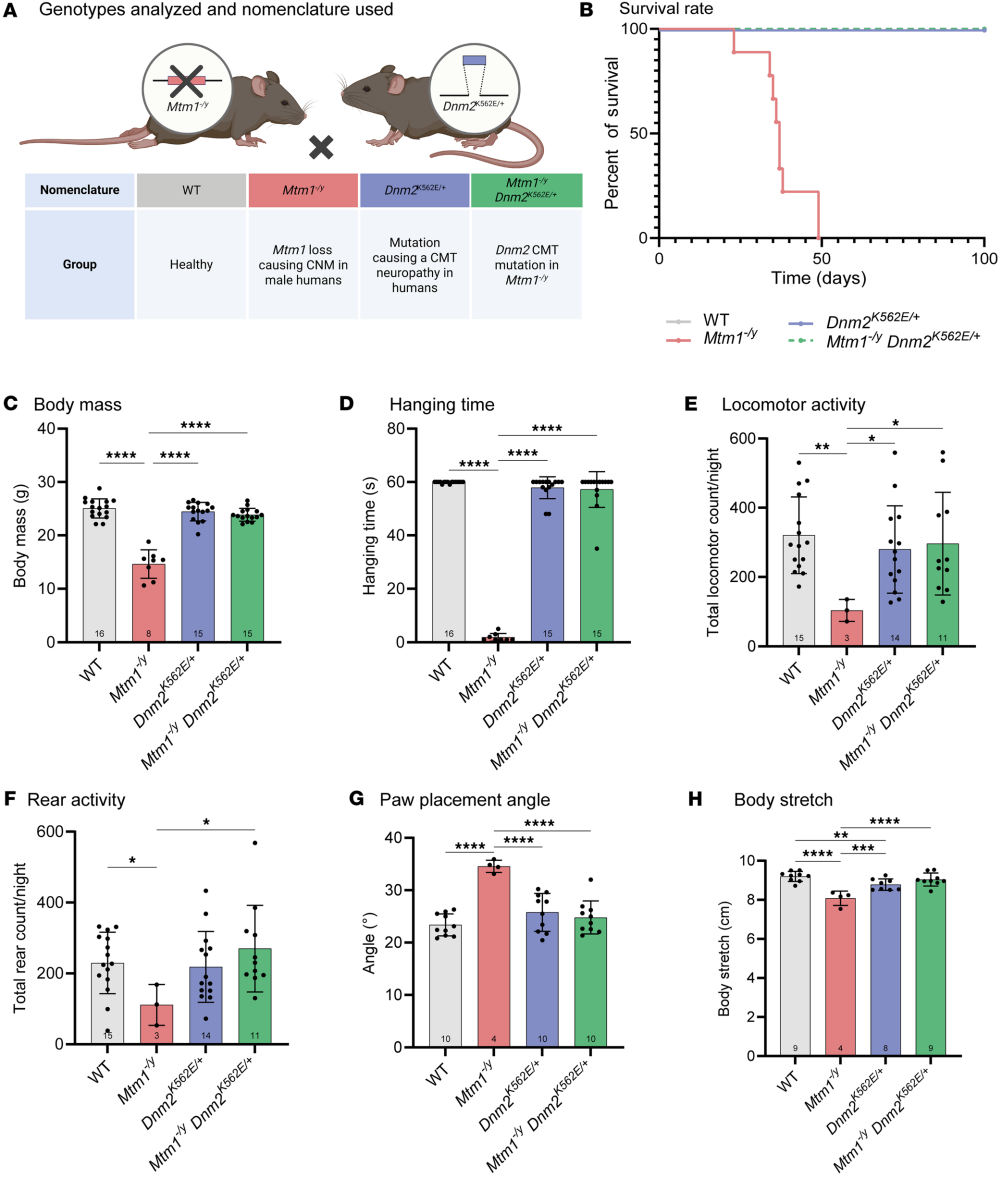
Systemic expression of the DNM2 mutant K562 restores whole-body motor performance in *Mtm1^–/y^* *Dnm2^K562E/+^* mice. (**A**) Mouse cohorts used for systemic proof of concept (males only). Created in BioRender (Goret M, 2026, https://BioRender.com/jqvfspw). (**B**) Survival curve of the 4 groups analyzed from birth to 100 days (8 ≤ *n* ≤ 10). (**C**) Body mass at 8 weeks (8 ≤ *n* ≤ 16). (**D**) Hanging test performance at 8 weeks; maximum hanging time = 60 seconds (8 ≤ *n* ≤ 16). (**E**) Total locomotor activity during the night; one count corresponds to one detected back-and-forth movement event in the cage (3 ≤ *n* ≤ 15). (**F**) Total rearing activity during the night (3 ≤ *n* ≤ 15). (**G**) Angle of the hind feet relative to the body axis during treadmill walking (4 ≤ *n* ≤ 10). (**H**) Body stretch (nose-to-tail base length) measured during treadmill walking at 8 weeks (4 ≤ *n* ≤ 9). Each dot represents 1 mouse. Values are shown as mean ± SD; *P < 0.05, **P < 0.01, ***P < 0.001, ****P < 0.0001. (**C**, **E**, **G**, and **H**) Ordinary 1-way ANOVA. (**D** and **F**) Kruskal-Wallis test.

While AAV-mediated expression allows rapid testing of DNM2 variants, it results in non-physiological overexpression that may confound phenotypic interpretation. To evaluate the effects of disrupting DNM2 lipid binding at physiological expression levels, we crossed *Mtm1^–/y^* XLCNM mice with *Dnm2^K562E/+^* CMT knockin mice to obtain *Mtm1^–/y^* *Dnm2^K562E/+^* double mutants. *Dnm2^K562E/+^* mice are a CMT model that expresses mutant lipid-binding-defective DNM2 systemically under its endogenous promoter and develop a primary myopathy characterized by mild muscle atrophy and fiber hypotrophy. Notably, mice expressing DNM2 K562E mutant and lacking the second *Dnm2* allele in Schwann cells (*Dnm2^K562E/SC–^*) develop a severe demyelinating neuropathy (51). We compared *Mtm1^–/y^* *Dnm2^K562E/+^* double mutants with *Mtm1^–/y^* mice to assess rescue, and with WT mice for potential normalization, at 8 weeks of age (Figure 5A). A subset of double mutants was maintained for lifespan analysis, which revealed a complete rescue compared with *Mtm1^–/y^* mice (Figure 5B), with the oldest animal reaching at least 80 weeks of age. In addition, body mass was normalized (Figure 5C), as were motor functions, including hanging test, actimetry, and gait analyses (Figure 5, D–H).

**Figure 6:**
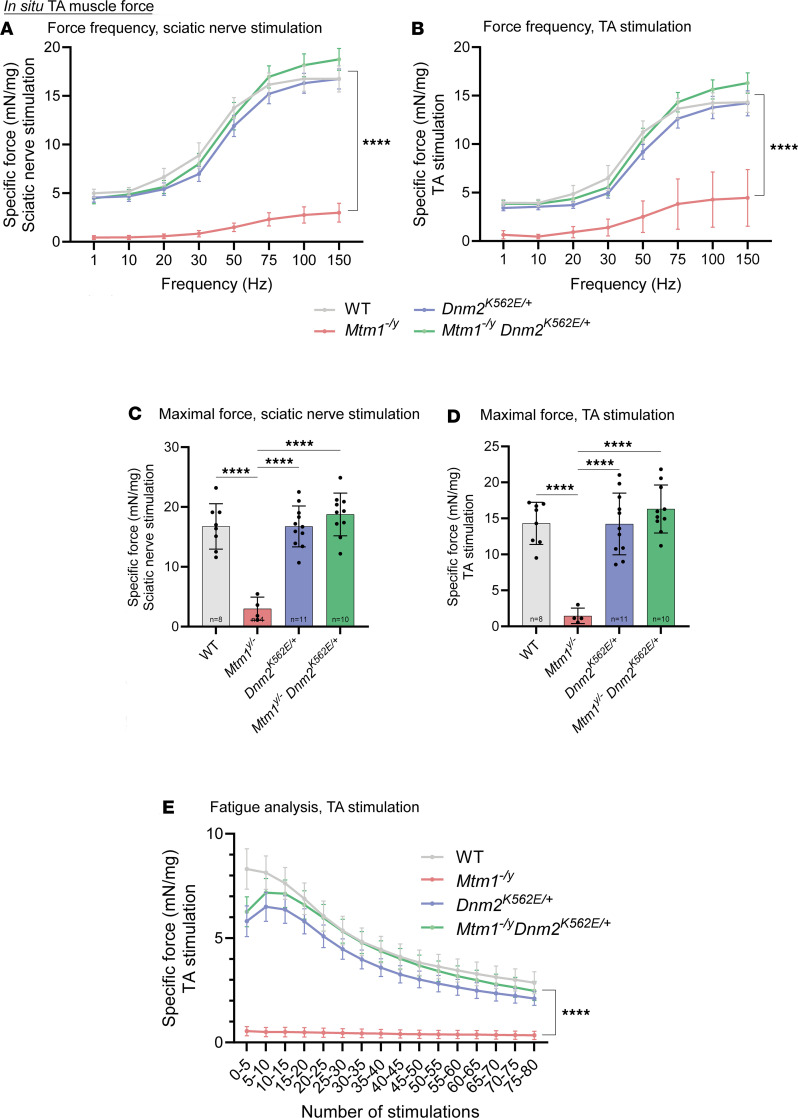
Systemic expression of the DNM2 mutant K562 restores in situ muscle force in *Mtm1^–/y^* *Dnm2^K562E/+^* mice. (**A** and **B**) TA specific force–frequency curve after stimulation of the sciatic nerve (4 ≤ *n* ≤ 11) (**A**) or the TA (**B**) at incremental frequencies (1–150 Hz) at 8 weeks, normalized to TA mass (4 ≤ *n* ≤ 11). (**C** and **D**) Maximal specific force developed by the TA after stimulation of the sciatic nerve (4 ≤ *n* ≤ 11) (**C**) or the TA itself (**D**) at 150 Hz, normalized to TA mass (4 ≤ *n* ≤ 11). (**E**) TA muscle fatigue over 80 consecutive TA stimulations at 40 Hz, normalized to TA mass (4 ≤ *n* ≤ 10). Each dot represents 1 mouse. Values are shown as mean ± SD; ****P < 0.0001. (**A**, **B**, and **E**) Two-way ANOVA. (**C** and **D**) Ordinary 1-way ANOVA.

Muscle force was strongly rescued: force-frequency curves demonstrated full correction of TA weakness in double mutants following both sciatic nerve and direct muscle stimulation (Figure 6, A and B). TA maximal specific force was also fully normalized with both types of stimulation (Figure 6, C and D). Fatigue analysis showed that *Mtm1^–/y^* muscles produced almost no force during repetitive stimulations. This defect was corrected in double mutants (Figure 6E).

**Figure 7:**
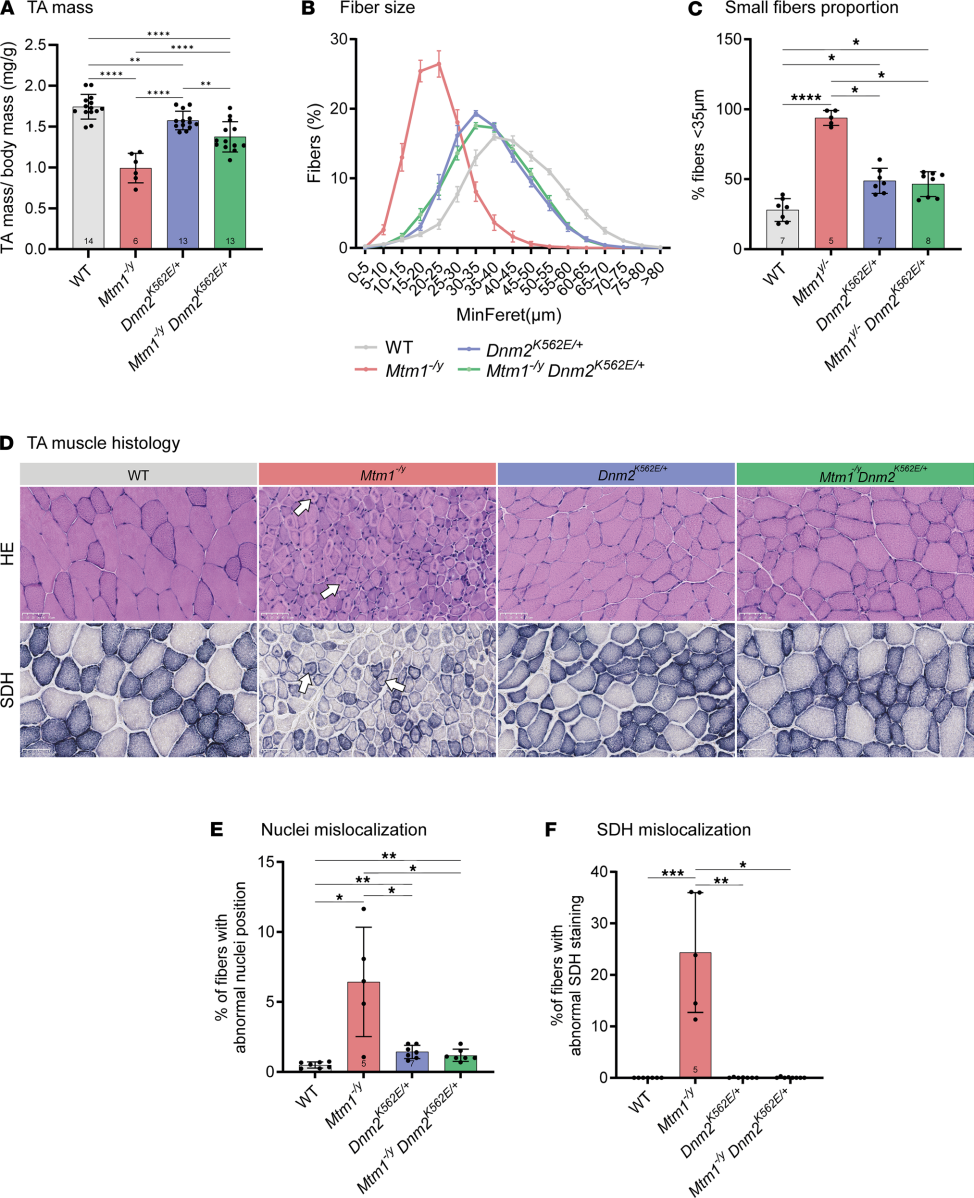
Systemic expression of the CMT neuropathy mutant K562 improves muscle atrophy and CNM histopathology in *Mtm1^–/y^* *Dnm2^K562E/+^* mice. (**A**) TA mass normalized to body mass at 8 weeks (6 ≤ *n* ≤ 14). (**B**) Distribution of TA fibers based on their MinFeret diameter (5 ≤ *n* ≤ 8). (**C**) Proportion of small fibers (MinFeret < 35 μm) in TA sections (5 ≤ *n* ≤ 8). (**D**) TA transversal sections stained with H&E and SDH. White arrows indicate mislocalization. Scale bars: 50 μm. (**E**) Percentage of myofibers with mislocalized nuclei (5 ≤ *n* ≤ 7). (**F**) Percentage of myofibers with abnormal SDH oxidative activity (5 ≤ *n* ≤ 8). Each dot represents 1 mouse. Values are shown as mean ± SD; *P < 0.05, **P < 0.01, ***P < 0.001, ****P < 0.0001. (**A**) One-way ANOVA. (**E**) Brown-Forsythe ANOVA. (**C** and **F**) Kruskal-Wallis test.

TA muscle mass was reduced in both *Mtm1^–/y^* CNM and, to a lesser extent, *Dnm2^K562E/+^* CMT mice compared with WT, with partial rescue observed in the double mutants compared with the CNM model (Figure 7A). A similar pattern was seen for fiber size: both models showed fiber hypotrophy, more pronounced in the CNM model, and the double mutants displayed partial rescue relative to CNM but no improvement compared with CMT (Figure 7, B and C). Histological analyses further revealed that organelle mispositioning was markedly improved in *Mtm1^–/y^* *Dnm2^K562E/+^* mice compared with *Mtm1^–/y^* mice, as shown by H&E and SDH staining and corresponding quantifications (Figure 7, D–F).

**Figure 8:**
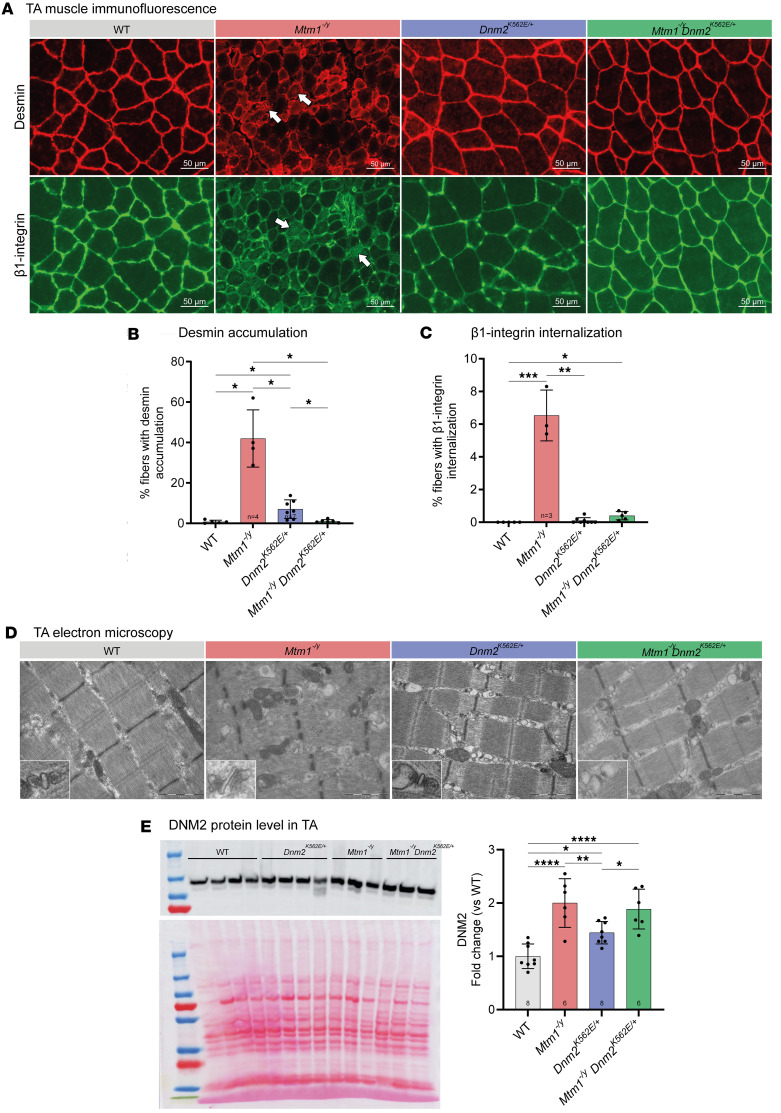
Systemic expression of the CMT neuropathy mutant K562 improves muscle organization in *Mtm1^–/y^* *Dnm2^K562E/+^* mice. (**A**) Immunolabeling of desmin (top) and β_1_-integrin (bottom) in transversal TA sections. Arrows indicate fibers with abnormal central accumulation of the staining. Scale bars: 50 μm. (**B** and **C**) Proportion of fibers with abnormal localization of desmin (4 ≤ *n* ≤ 7) (**B**) and β_1_-integrin (**C**) in TA sections at 8 weeks (3 ≤ *n* ≤ 8). (**D**) Electron microscopy images of longitudinal TA sections. Scale bars: 1 μm. Zoomed views of triads in bottom-left corners (×8 relative to main image). (**E**) Representative Western blot and quantification of DNM2 protein in TA (6 ≤ *n* ≤ 8). Each dot represents 1 mouse. Values are shown as mean ± SD; *P < 0.05, **P < 0.01, ***P < 0.001, ****P < 0.0001. (**B**) Brown-Forsythe ANOVA. (**C**) Kruskal-Wallis test. (**E**) Ordinary 1-way ANOVA.

To assess the molecular and structural mechanism of the rescue, transversal sections of TA muscle were analyzed by immunofluorescence. Abnormal desmin accumulations were detected in both single-mutant models, consistent with previous reports (45, 47, 52), and were fully normalized in the double mutants (Figure 8, A and B). Similarly, β_1_-integrin accumulations observed in *Mtm1^–/y^* fibers were largely ameliorated in the double mutants, although the change was not statistically significant (Figure 8, A and C).

Ultrastructural analysis revealed that *Mtm1^–/y^* fibers were severely disorganized, with centrally located nuclei, dilated sarcoplasmic reticulum (SR), and abnormal triads and T tubules, while mitochondria appeared small but structurally intact (Figure 8D). SR dilatation and disorganized triads/T tubules were observed in *Dnm2^K562E/+^* fibers, though mitochondria remained normal. In the *Mtm1^–/y^* background, K562E expression partially improved these defects but did not restore to WT-like structure: SR dilatation and triad/T tubule abnormalities persisted, and some fibers showed mitochondrial degeneration.

Since DNM2 levels are elevated in *Mtm1^–/y^* muscle and normalization of DNM2 levels has been linked to phenotype rescue (32, 33), we quantified protein levels by Western blot. DNM2 was approximately 2-fold higher in *Mtm1^–/y^* mice and 1.4-fold higher in *Dnm2^K562E/+^* mice compared with WT (Figure 8E). In double mutants, DNM2 remained elevated (~1.9-fold vs. WT), indicating that phenotypic rescue occurred independently of DNM2 level normalization.

In conclusion, these results demonstrate that sustained genetic inhibition of DNM2 lipid binding is sufficient to rescue the majority of phenotypes in the severe *Mtm1^–/y^* CNM model and significantly prolong lifespan, whereas the reciprocal rescue of the *Dnm2^K562E/+^* phenotype by MTM1 loss does not occur (Supplemental Figure 7). In contrast, crossing *Mtm1^–/y^* mice with mice expressing hyperactive DNM2 mutants (S619L or R465W) exacerbated the disease, with few or no pups surviving after birth (Supplemental Figure 8).

## Discussion

In this study, we dissected the contribution of distinct DNM2 functions to muscle pathology by expressing a panel of DNM2 variants in WT and *Mtm1^–/y^* muscles. Our findings reveal that lipid binding emerges as the critical determinant of both disease pathology and therapeutic rescue. Furthermore, genetic inhibition of lipid binding from embryogenesis in *Mtm1^–/y^* rescued most phenotypes.

### Role of DNM2 functions in CNM pathology.

Exogenous expression of WT DNM2 or loss-of-function mutants in WT muscle generally induced CNM-like phenotypes, likely reflecting the deleterious effects of increased DNM2 levels. Previous studies showed that increasing WT DNM2 level through transgenesis or AAV transduction induced CNM-like phenotypes as well (31, 53). Here we observed that injecting loss-of-function DNM2 mutants disrupted DNM2 distribution and impaired membrane trafficking, remodeling, and protein homeostasis, likely affecting organelle positioning, fiber morphology, and ultrastructure and ultimately altering muscle force. Notably, the distinct mutants produced different phenotypic profiles.

Nuclear mislocalization, a hallmark of CNM, was induced strongly in WT muscle by WT, K44A, K142A, and KARW (>30% fibers) and to a lesser extent by ΔPRD and R399A (<15% fibers) (Figure 1D). In *Mtm1^–/y^* muscle, where nuclei are already mislocalized, most mutants, except K562E (defective in lipid binding; refs. 15, 19) and ΔPRD (lacking protein-protein interactions), further exacerbated nuclear positioning defects (Figure 3F). In agreement, the *Dnm2^K562E/+^* mouse model does not display a CNM histological phenotype nor nucleus misposition in muscle. K562E overexpression following injection of WT muscle did not cause CNM histopathology or muscle atrophy (36). In line with these observations, we hypothesize that nuclear mislocalization requires lipid binding but not GTPase activity. Transposed to CNM mouse models and patients, these data suggest that *DNM2* mutations cause CNM by increasing DNM2 level or lipid binding. Indeed, DNM2 level is increased in the *Dnm2^S619L/+^* and *Dnm2^R369W/+^* mouse models (38, 48). More generally, our data suggest that lack of MTM1 function, as found in *MTM1*-CNM patients and the *Mtm1^–/y^* mouse, would also cause CNM through increasing DNM2 level or lipid binding. We previously reported a significant increase in DNM2 protein level in both *MTM1*-CNM patient muscles and the *Mtm1^–/y^* mouse (32); however, the link between MTM1 activity and DNM2 lipid binding remains unclear.

GTPase activity, on the other hand, seems crucial for membrane trafficking but less related to the CNM pathomechanism. Mutants impairing this function, including KARW, K44A, and K142A, and pharmacological inhibition or DNM2 depletion, led to defects in endosome maturation and autophagosome homeostasis in cultured cells (12, 54). The fact that these mutants induced a CNM-like phenotype when overexpressed in WT mice supports that an increase in GTPase activity is not the main cause of CNM.

Despite its low effect on nuclei mislocalization, ΔPRD caused the largest reductions in muscle force and TA mass, indicating that centralized nuclei alone are not the primary determinant of contractile dysfunction, consistent with prior observations in DNM2 overexpression models (55). All DNM2 functions, however, appear necessary to maintain triad structure, as defects were observed here across mutants affecting lipid binding, oligomerization, protein interactions, or GTPase activity.

The R399A mutant, which stabilizes DNM2 in a dimeric cytosolic state, induced profound intracellular disorganization and a strong decrease in oxidative activity linked to mitochondria without major effects on nuclear positioning. Importantly, these induced phenotypes are different from CNM hallmarks.

### Lipid binding of DNM2 as a key therapeutic target.

It has previously been shown that reducing DNM2 protein level has a therapeutic effect in several *Mtm1*- or *Dnm2*-CNM mouse models (32, 34, 35, 56). Here we wanted to explore how targeting specific DNM2 protein functions could improve CNM-related phenotypes.

In *Mtm1^–/y^* muscles, only the expression of the lipid-binding-defective K562E mutant consistently improved muscle force and mass, histology, sarcomere organization, and protein localization. Other mutants failed to rescue or even aggravated pathology, likely because most other mutants have a WT lipid-binding property and because DNM2 overexpression itself is deleterious. By contrast, overexpression of K562E produced minimal defects in WT muscle, suggesting a therapeutic candidate in the CNM context. This contrasts with the muscle phenotype reported in *Dnm^K562E/+^* mice (51), likely reflecting differences between acute overexpression in adult muscle and endogenous expression from embryogenesis. The therapeutic relevance of lipid binding was further demonstrated using *Mtm1^–/y^ Dnm2^K562E/+^* mice, which showed normalized lifespan, motor function, and muscle morphology. This supports that decreasing DNM2 lipid binding during embryogenesis and in the long term is not detrimental in a CNM context. Notably, these improvements occurred despite persistently elevated DNM2 protein levels, indicating that selective inhibition of lipid binding, rather than global protein reduction, is sufficient for phenotypic correction. This aligns with a prior report showing that K562E mutation alleviates CNM phenotype in a *DNM2*-related mouse model (45). Mechanistically, we hypothesize that reducing DNM2-lipid interactions may restore membrane trafficking and protein homeostasis, thereby mitigating disease progression.

Taken together, our findings highlight lipid binding as the key driver of CNM pathology and provide a proof of concept that selective reduction of this activity is sufficient to ameliorate X-linked CNM disease manifestations in vivo. Modulating other DNM2 functions appears to be either insufficient or deleterious, emphasizing the need for strategies that selectively inhibit DNM2-lipid interactions. Future approaches could include small molecules targeting the PH domain or allosteric modulators that limit membrane association while preserving other DNM2 functions.

## Methods

### Sex as a biological variable.

Only male mice were used. Myotubular myopathy is an X-linked recessive disease (*MTM1* mutations) that primarily affects males.

### Materials.

pAAV plasmids containing full-length human DNM2 cDNA (NCBI Reference Sequence NM_001005360.2) were generated as previously described (31) and the different mutations introduced by primer-directed PCR mutagenesis. All constructs were verified by Sanger sequencing.

The following primary commercial antibodies were used: anti-dysferlin (Abcam; ab15108), anti-desmin (Abcam; ab152000), and anti–β_1_-integrin (Chemicon; MAB1997). Mouse anti–β-actin and rabbit anti-DNM2 antibodies (R2680, R2865) (31) were made on-site at the antibody facility at the IGBMC. Alexa-conjugated secondary antibodies were purchased from Invitrogen. Secondary antibodies against mouse and rabbit IgG, conjugated with horseradish peroxidase, were purchased from Jackson ImmunoResearch Laboratories. Hoechst nuclear stain was from Sigma-Aldrich. To detect neuromuscular junctions (NMJs), α-Bungarotoxin CF488A Conjugate (Interchim; 00005) was used.

The following products were purchased: ECL chemiluminescent reaction kit (Thermo Fisher Scientific; 34577), FluorSave reagent (Merck; 345789), DC Protein Assay (Bio-Rad; 5000111), and Marker Reducing Buffer (Thermo Fisher Scientific; 39000).

### Production and purification of AAV.

Recombinant adeno-associated virus (AAV2/1) was generated as described before (31, 36). Briefly, AAVs were generated by triple transfection of HEK293T cell line with pAAV2 containing DNM2 insert under the control of the CMV promoter and flanked by serotype 2 inverted terminal repeats, pHelper coding the adenovirus helper functions, and pXR1 containing rep and cap genes of AAV serotype 1. AAV vectors were harvested from cell lysate 48 hours after transfection lysate and purified by iodixanol gradient ultracentrifugation (OptiPrep) followed by dialysis and concentration against Dulbecco’s phosphate-buffered saline (DPBS) using centrifugal filters (Amicon Ultra-15 Centrifugal Filter Devices 100K, Millipore). Viral titers were quantified by qPCR using the LightCycler480 SYBR Green I Master mix (Roche) and primers targeting CMV enhancer sequence. Titers are expressed as viral genomes per milliliter (vg/mL).

### Animals.

*Mtm1^–/y^* (129/SvPAS) mice were previously generated and characterized (33, 49). *Mtm1^–/y^* *Dnm2^K562E/+^* mice were obtained by breeding of female heterozygous *Mtm1*^+/–^ mice with male heterozygous *Dnm2^K562E/+^* mice (C57BL/6J, previously generated and characterized; refs. 45, 51). Male *Mtm1^+/y^* *Dnm2^K562E/+^* (*Dnm2^K562E/+^*) and *Mtm1^+/y^* *Dnm2^+/+^* (WT) littermates were used as controls because myotubular myopathy is an X-linked recessive disease that predominantly affects males. The number of animals used for each experiment is reported in Supplemental Table 1.

Finger biopsies were used for genotyping. The floxed *Dnm2* allele was identified using the forward GCCATCTTCAACACAGAGCAGAGGTG and reverse TACACTGTCTGCACTGTCGAGCCCTG primers for the K562E mutation. Forward AGCACATGGGAGGTTGAG and reverse GGCTTTAACCAAGGATTCACA primers were used for *Mtm1* genotyping.

Animals were housed in cages with access to food ad libitum and in a temperature-controlled room (19°C–22°C) with a 12-hour light/12-hour dark cycle. Mice were euthanized by cervical dislocation and humanely sacrificed when required according to national and European legislation on animal experimentation.

### Mouse phenotyping.

Behavioral tests were conducted at 8 weeks of age, in the same order for all animals, by a single experimenter blinded to genotype and injection. The hanging test measured latency to fall from an inverted grid (maximum 60 seconds); 3 trials were performed per animal, and the 2 best were averaged. Gait analysis was performed on a motorized treadmill equipped with a camera, at a constant speed (8 cm/s). Body stretch (nose to tail base when hind paw was in contact with the surface) and paw placement angle (angle between body axis and paw center when in contact with the surface) were quantified. Three measures were averaged for body stretch and 6 for paw angle (3 per side). The actimetry test measured spontaneous locomotor activity in mice using a closed cage equipped with infrared light beams. It tracked the number of rears and locomotor activity, recorded during the night from 7 pm to 7 am, after a period of acclimatization from 11 am to 7 pm. Activity counts were averaged over the night.

### AAV transduction of tibialis anterior muscle.

AAV2/1 was injected in the tibialis anterior (TA) of 3-week-old male WT and *Mtm1^–/y^* 129/SvPAS mice, as previously described (36). Mice were anesthetized by intraperitoneal injection of a ketamine (20 mg/mL) and xylazine (0.4%) solution at 5 μL/g body mass. Fifteen microliters of 1 × 10^11^ vg/mL AAV2/1 encoding either WT DNM2, DNM2 mutants, or empty AAV vector (control) were injected randomly into the left or right TA muscles; injections were not paired within individual animals. Mice were sacrificed 2 weeks after injection.

### In situ muscle force measurement.

The contractile properties of the TA muscle were measured in response to either sciatic nerve or direct muscle stimulation using the in situ Whole Animal System 1305A (Aurora Scientific). Mice were anesthetized with intraperitoneal injections of medetomidine/fentanyl mix (2/0.28 mg/kg), diazepam (8 mg/kg), and fentanyl (0.28 mg/kg). The distal TA tendon was partially excised and attached to an isometric transducer (Aurora Scientific). The sciatic nerve was exposed through a small thigh incision. Isometric tetanic force was first recorded following sciatic nerve stimulation (150 Hz, 0.5 seconds), followed by a force-frequency analysis (1–150 Hz). The same protocol was repeated with direct muscle stimulation. Fatigue was assessed using 80 consecutive tetanic stimulations (40 Hz, 1 second on/3 seconds off), and tetanic force was averaged every 5 contractions. Fatigue was expressed as the percentage drop in force from the first 5 to the last 5 contractions.

Specific force was calculated either by division of maximal force by muscle cross-sectional area (mN/mm^2^), derived from wet muscle mass, optimal muscle length, and muscle density (1.06 mg/mm^3^); or by division of maximal force by muscle mass (mN/mg).

### Protein extraction and Western blot.

TA muscles were lysed in RIPA buffer supplemented with 1 mM PMSF, 1 mM DTT, and cOmplete Mini EDTA-free protease inhibitor cocktail (Roche; 11836170001) using a Precellys tissue homogenizer (Bertin Technologies). Protein concentrations were determined with the DC Protein Assay. Samples were denatured at 95°C for 5 minutes in 5× Lane Marker Reducing Buffer (Thermo Fisher Scientific; 39000). Then 10 μg of protein was loaded and separated in 8% or 10% SDS-PAGE gel and transferred on nitrocellulose membrane using a Trans-Blot Turbo Transfer System (Bio-Rad). Membranes were stained with Ponceau S, then blocked for 1 hour in Tris-buffered saline containing 5% nonfat dry milk and 0.1% Tween 20 before an incubation overnight with primary antibody DNM2 2865 (1:500–1:1,000; homemade anti-proline rich domain), DNM2 2680 (1:200; homemade anti–pleckstrin homology/GTPase effector domain), or β-actin (1:1,000; homemade) diluted in blocking buffer containing 5% milk. Membranes were subsequently incubated for 1 hour at room temperature with anti-mouse or anti-rabbit secondary antibodies coupled with horseradish peroxidase (1:5,000–1:10,000; Jackson ImmunoResearch Laboratories). Nitrocellulose membranes were visualized in an Amersham Imager 600 (GE Healthcare Life Sciences) using an ECL chemiluminescent reaction kit (Pierce). Band intensity was determined by densitometry using ImageJ (NIH). All original, uncropped Western blot images used for quantification, including those not shown in the figures and their corresponding loading controls, are available in the supplementary material.

### Histology.

TA muscles were snap-frozen in liquid nitrogen–cooled isopentane and stored at –80°C. Eight-micrometer transversal cryosections were prepared and stained for hematoxylin and eosin (H&E) and succinate dehydrogenase (SDH) histology analysis. Images of the entire muscle sections were acquired with a Hamamatsu NanoZoomer 2HT slide scanner. Myofiber segmentation was performed in H&E sections using the Cellpose algorithm (57). The minimum Feret diameter (MinFeret) was calculated using Fiji image analysis software (58), and the number of fibers with mislocalized (centralized or internalized) nuclei was manually counted using the Fiji Cell Counter plug-in.

### Electron microscopy.

Transmission electron microscopy (TEM) was carried out on pieces of TA muscles fixed in 2.5% paraformaldehyde and 2.5% glutaraldehyde (Electron Microscopy Sciences) in 0.1 M cacodylate buffer (pH 7.4). Muscles were fixed in 1% osmium tetroxide (OsO_4_) in H_2_O at 4°C. Then samples were dehydrated with increasing concentrations of ethanol, and embedded with a graded series of epoxy resin. Samples were finally polymerized at 60°C for 48 hours. Ultrathin sections of 70 nm were obtained and stained with uranyl acetate and lead citrate. They were observed by Philips CM12 TEM electron microscope (Philips, FEI Electron Optics, Eindhoven, Netherlands) operated at 80 kV and equipped with an Orius 1000 CCD camera (Gatan).

### Immunostaining of muscle sections.

Isopentane-frozen muscles were sectioned transversally (8 μm) and fixed with 4% paraformaldehyde before immunostaining. Sections were permeabilized with 0.5% PBS–Triton X-100 and blocked with 5% BSA in PBS. Primary antibodies (see *Materials*) were diluted in 1% BSA as follows: DNM2 R2865 (1:100), dysferlin (1:50), desmin (1:100), and β_1_-integrin (1:50). Alexa Fluor 488–, 555–, 594–, or 647–conjugated secondary antibodies (anti-rabbit or anti-rat) were used at 1:250 in 1% BSA. For longitudinal immunostaining, hind limbs were fixed in 4% paraformaldehyde for 24 hours. The TA muscle was then dissected, cryoprotected in 30% sucrose, and stored at –20°C before sectioning (8 μm). NMJs were labeled with α-Bungarotoxin CF488A Conjugate (1:1,000). Images for AAV-injected muscles were acquired using a Leica SP8-UV confocal microscope (Leica Microsystems), and *Z*-projections are shown. Images from genetic crosses were acquired using a Hamamatsu NanoZoomer 2HT slide scanner. NMJ fragmentation and circularity were quantified using Fiji. For circularity, a value of 1 indicates a perfect circle, and values approaching 0 indicate increasingly elongated structures.

### Statistics.

All statistical analyses and graph generation were performed using GraphPad Prism. Normality was assessed using the Shapiro-Wilk test. For normally distributed data with equal variances, 1-way ANOVA followed by uncorrected Fisher’s least significant difference test was used for pairwise comparisons. When variances were unequal, Brown-Forsythe and Welch’s ANOVA was performed, followed by 2-tailed Welch’s *t* tests. For non-normally distributed data, the Kruskal-Wallis test was used, followed by uncorrected Dunn’s test. For DNM2-mutant injections, only statistically significant differences relative to the empty control group are shown. For genetic crosses, all pairwise comparisons were performed, and only statistically significant differences are displayed. *P* values less than 0.05 were considered significant. Graphs display individual data points with mean ± SD. A detailed summary of the statistical tests and group sizes is provided in Supplemental Table 1.

### Study approval.

All animal procedures were conducted in compliance with French and European regulations and were approved by the Com’Eth IGBMC–Institut Clinique de la Souris institutional ethics committee (Illkirch, France) under authorization numbers APAFIS 5640-2016061019332648, 21789-2019082616218526, 22713-2019103108289018, and 33431-2021101219473414.

### Data availability.

All source data supporting the findings of this study are provided in the Supporting Data Values file.

## Author contributions

JL designed the study. BSC and JL supervised the study and secured funding. RGO, XMM, and DR performed in vivo phenotyping, muscle force measurements, histology, immunofluorescence, and molecular analyses. RGO and XMM contributed to injections, electron microscopy acquisition, and project management. DR carried out genetic crosses. JDCN carried out injections and histology quantification. ND performed immunofluorescence. RSR conducted muscle force measurements. RGO and XMM wrote the initial draft, and MG prepared figures and wrote the final manuscript together with JL.

## Conflict of interest

JL and BSC patented DNM2 inhibition for the treatment of centronuclear myopathy (WO2015055859A1).

## Funding support

- Interdisciplinary Thematic Institute (ITI) IMCBio, as part of the ITI 2021–2028 program of the University of Strasbourg, CNRS, and INSERM.
- IdEx Unistra (grant ANR-10-IDEX-0002), the SFRI-STRATUS project (ANR-20-SFRI-0012), and EUR IMCBio (ANR-17-EURE-0023) under the framework of the France 2030 Program.
- Agence National de la Recherche (Dynather, ANR-18-CE17-0006-02).
- Cifre (ANRT, 2017/1270, to RGO).
- IGBMC International PhD Program (to XMM).
- Fondation Recherche Médicale fellowship (PLP20170939073 to RSR).

## Supplementary Material

Supplemental data

Unedited blot and gel images

Supplemental table 1

Supporting data values

## Figures and Tables

**Table 1:**
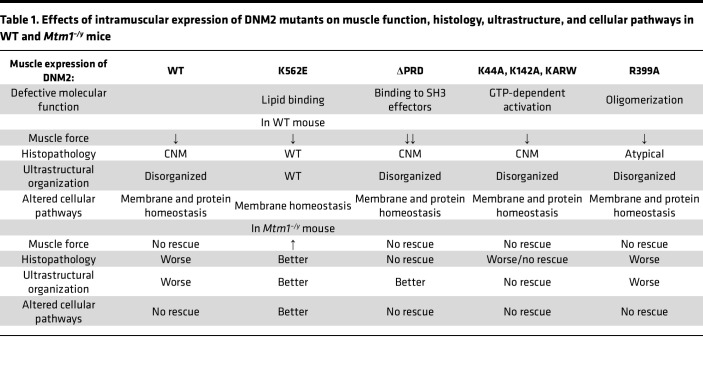
Effects of intramuscular expression of DNM2 mutants on muscle function, histology, ultrastructure, and cellular pathways in WT and *Mtm1^–/y^* mice

## Version 1 05/08/2026

Electronic publication
