# Supplementary material for: DNM2 lipid binding drives centronuclear myopathy and represents a potential therapeutic target
Source: JCI Insight. 2026 May 8;11(9):e204423. doi: 10.1172/jci.insight.204423 (PMC13232018; doi:10.1172/jci.insight.204423)
Supplement: Supplemental data [file jciinsight-11-204423-s207.pdf]

# Supplementary figures

# DNM2 lipid binding drives centronuclear myopathy and represents a potential therapeutic target

Raquel Gomez-Oca, Xènia Massana- Muñoz, David Reiss, Juliana De Carvalho Neves, Nadege Diedhiou, Roberto Silva-Rojas, Belinda S. Cowling, Marie Goret, Jocelyn Laporte

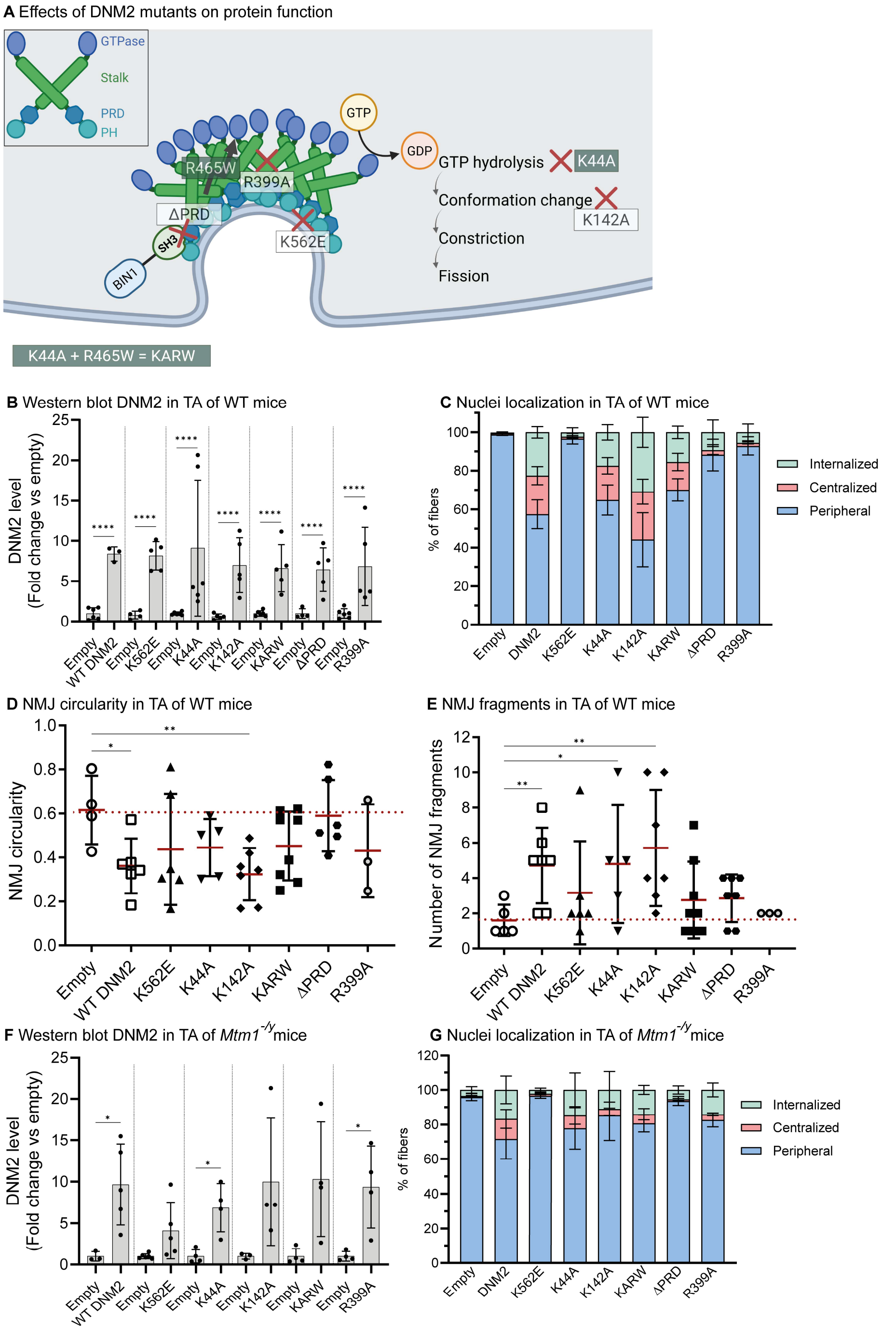


**Supplementary Fig. 1. Additional data on injected DNM2 mutants. (A)** Theoretical model of DNM2 function in membrane fission, placing the mutated isoforms analyzed in this study. DNM2 domains are detailed in the dimer representation (upper-left corner). Created in BioRender. Goret, M. (2026) https://BioRender.com/ezxeirw. **(B)** DNM2 protein quantification in TA after AAV injection, normalized to β-actin depicted in supplementary material (3≤n≤6). **(C)** Percentage of myofibers with central, internal or peripheral nuclei in injected WT TA (4≤n≤5). **(D)** NMJ circularity (0 corresponding to a line and 1 to a circle) in longitudinal TA sections (n = 3-8 NMJs from 1 mouse). **(E)** Number of fragments per NMJ in longitudinal TA sections (n = 3-8 NMJs from 1 mouse). **(F)** DNM2 protein quantification in *Mtm1^-/y^* TA after AAV injection, normalized to β-actin depicted in supplementary material (3≤n≤6). **(G)** Percentage of myofibers with central, internal or peripheral nuclei in injected *Mtm1^-/y^* TA (3≤n≤5). (B, F) Each dot represents one mouse. (D, E) Each dot represents one NMJ. Values are shown as mean ± SD, *p<0.05, **p<0.01, ****p<0.0001. (B, D, F) ANOVA. (E) Kruskal-Wallis.

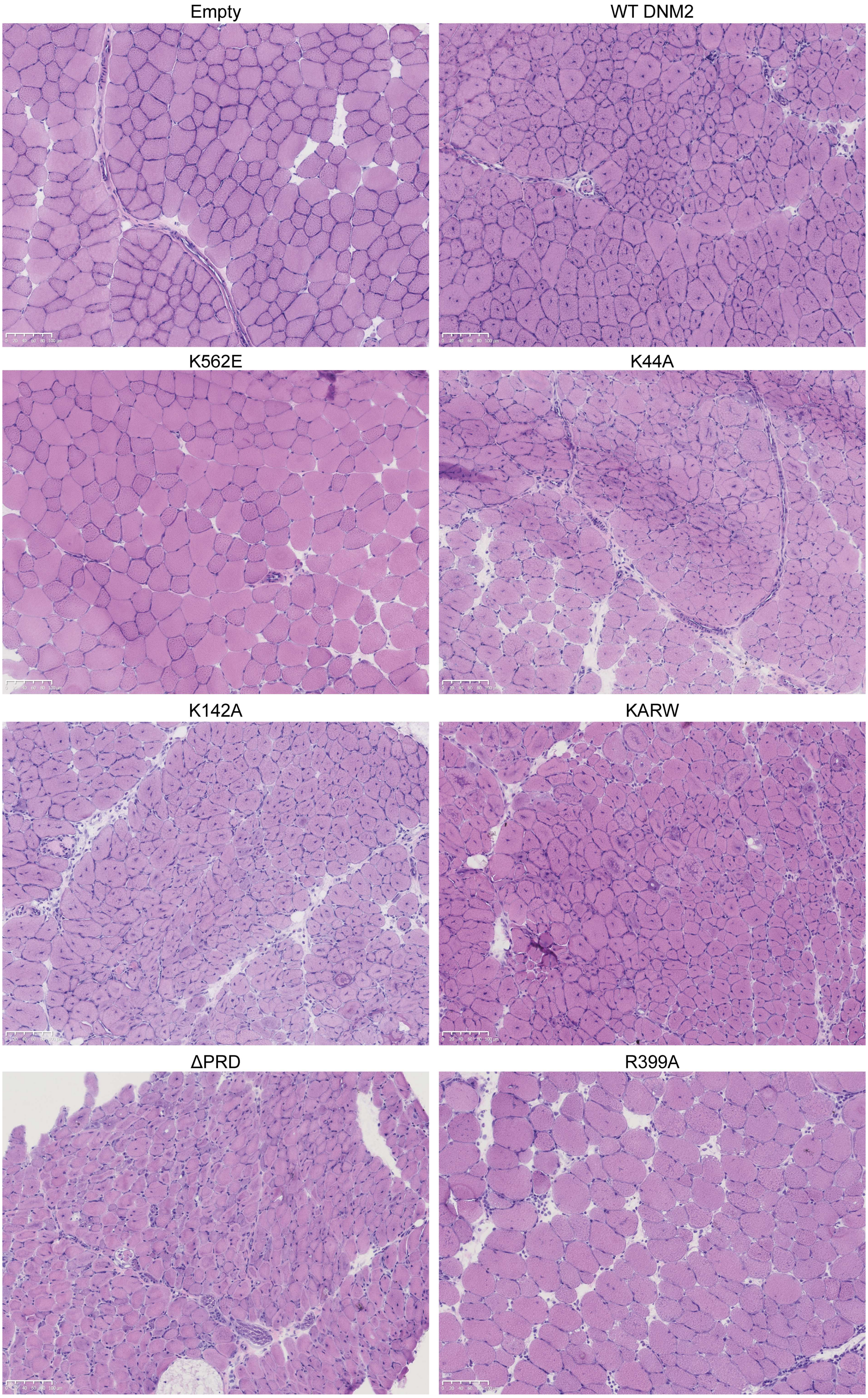


**Supplementary Fig. 2.** Transversal sections of TA muscles from WT mice injected with DNM2 variants, stained with hematoxylin and eosin. Scale bar = 100 µm.

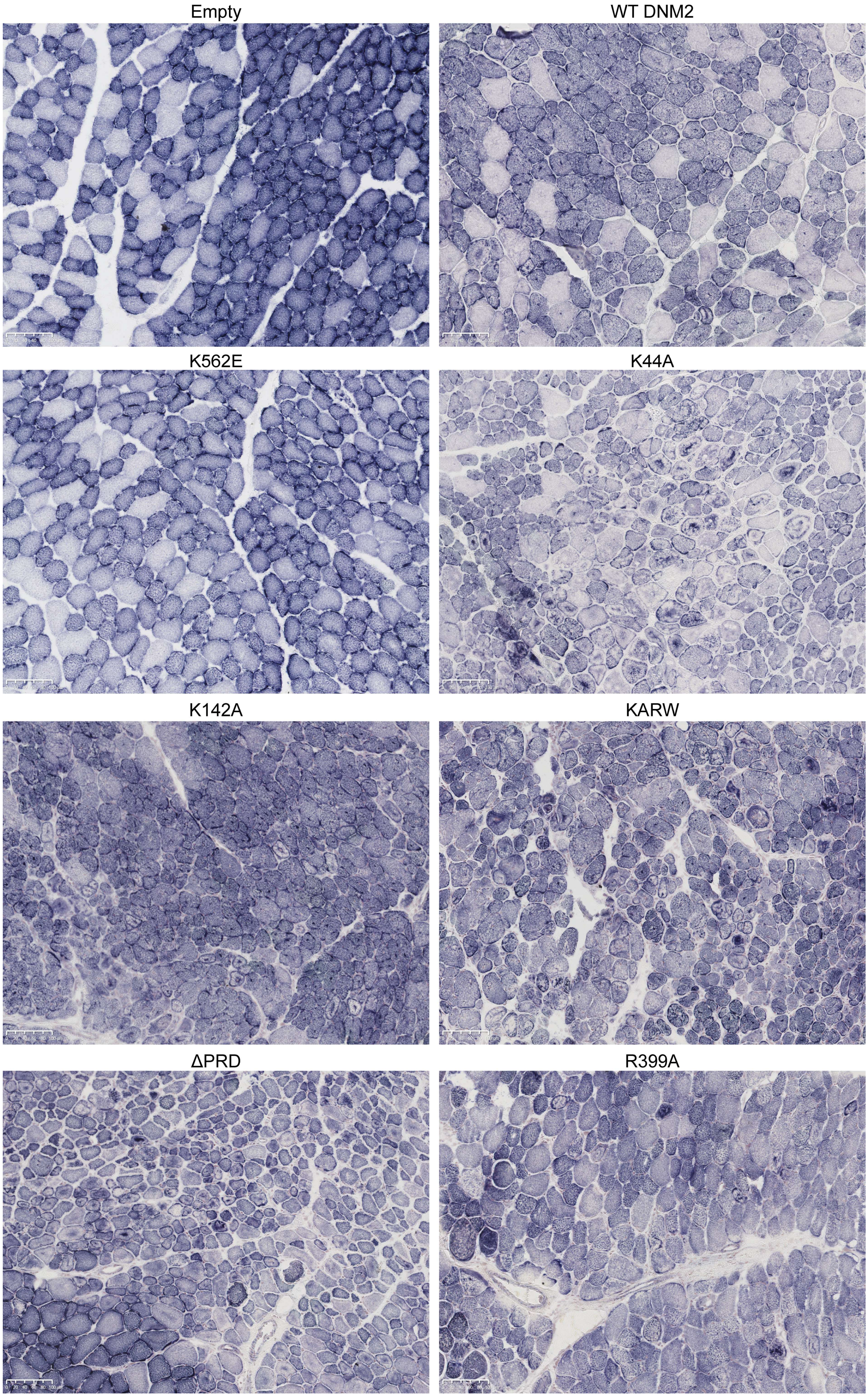


**Supplementary Fig. 3.** Transversal sections of TA muscles from WT mice injected with DNM2 variants, stained for succinate dehydrogenase activity. Scale bar = 100 µm.

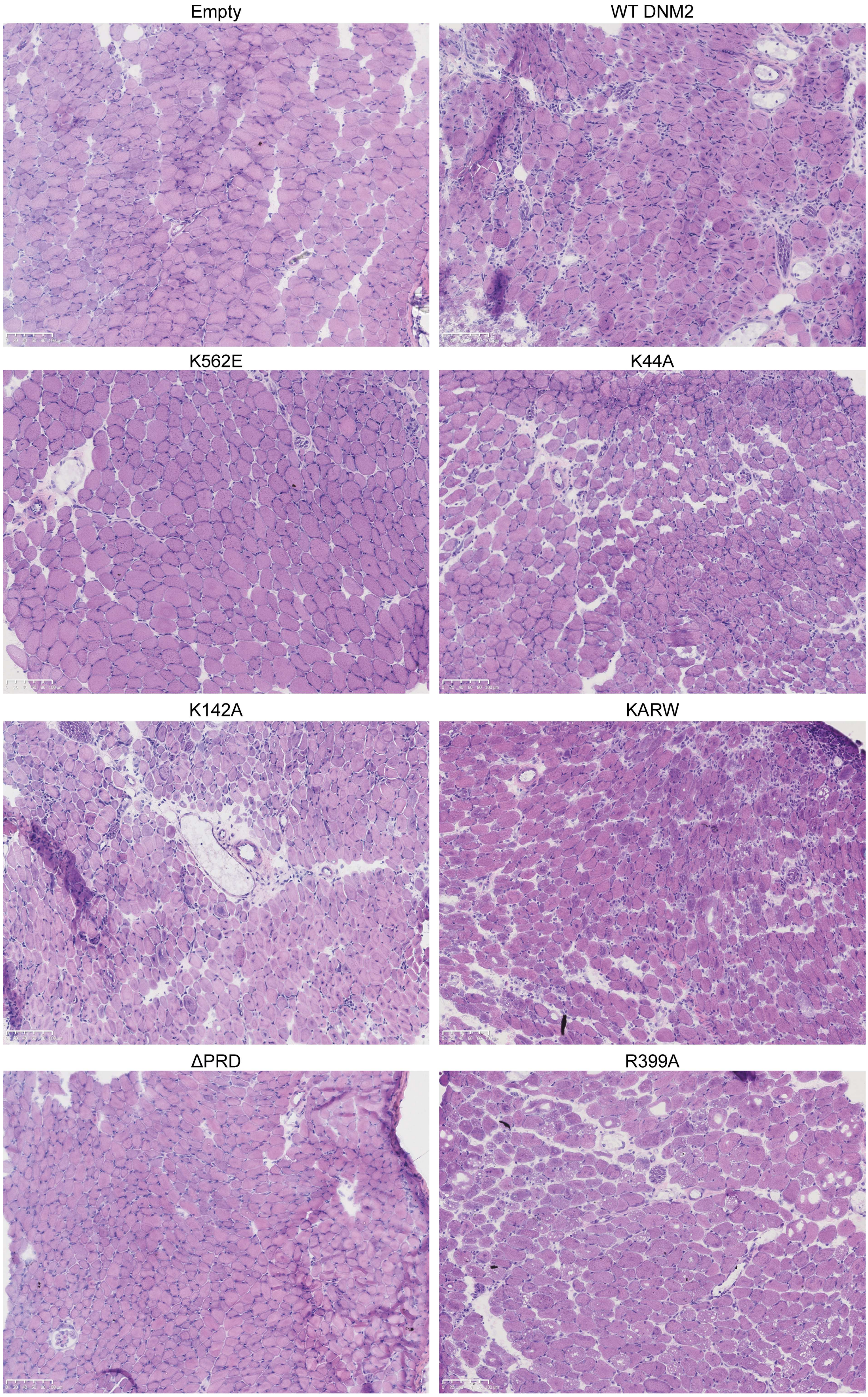


**Supplementary Fig. 4.** Transversal sections of TA muscles from *Mtm1*$^{-/y}$ mice injected with DNM2 variants, stained with hematoxylin and eosin. Scale bar = 100 µm.

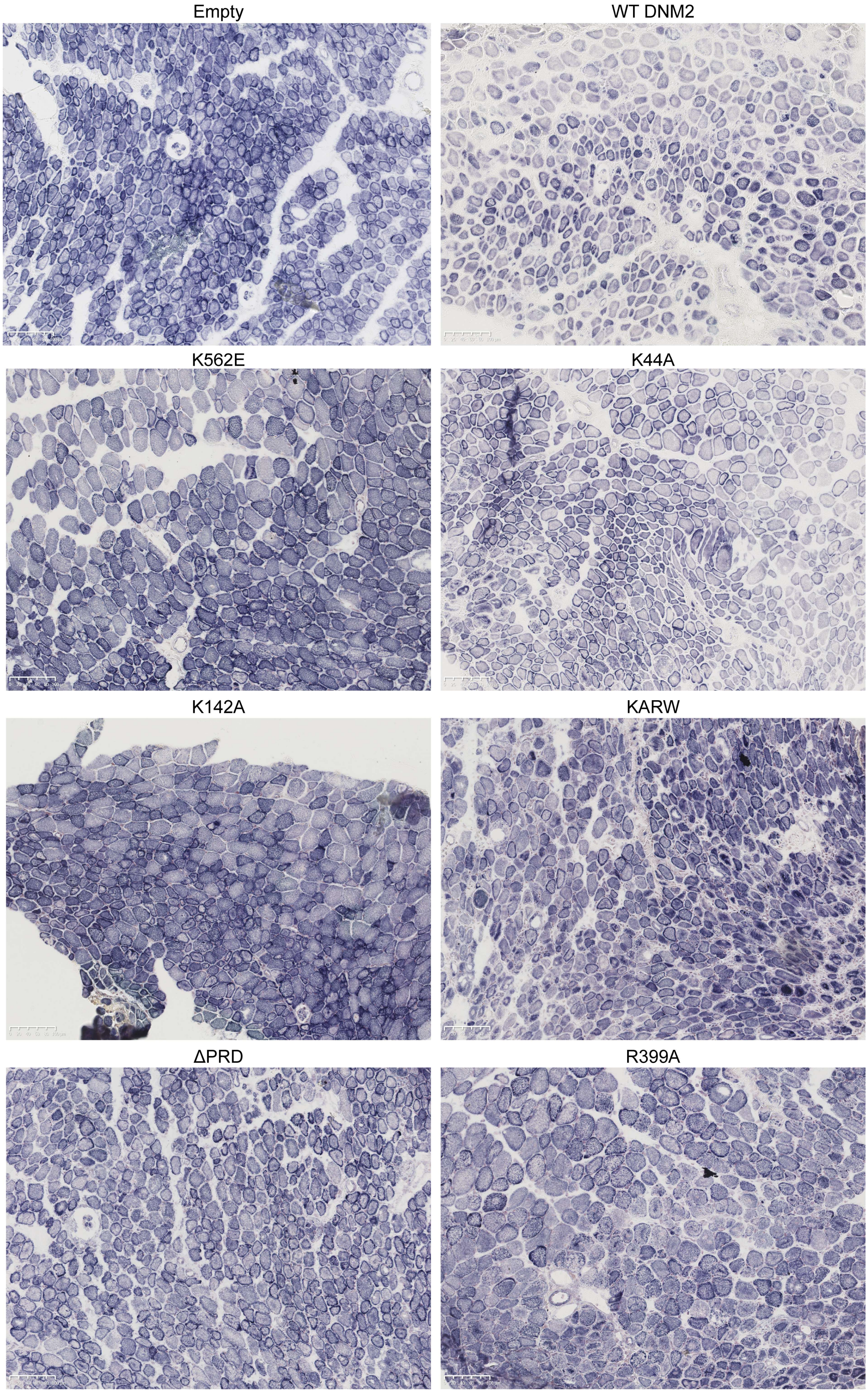


**Supplementary Fig. 5.** Transversal sections of TA muscles from *Mtm1*$^{-/y}$ mice injected with DNM2 variants, stained for succinate dehydrogenase activity. Scale bar = 100 µm.

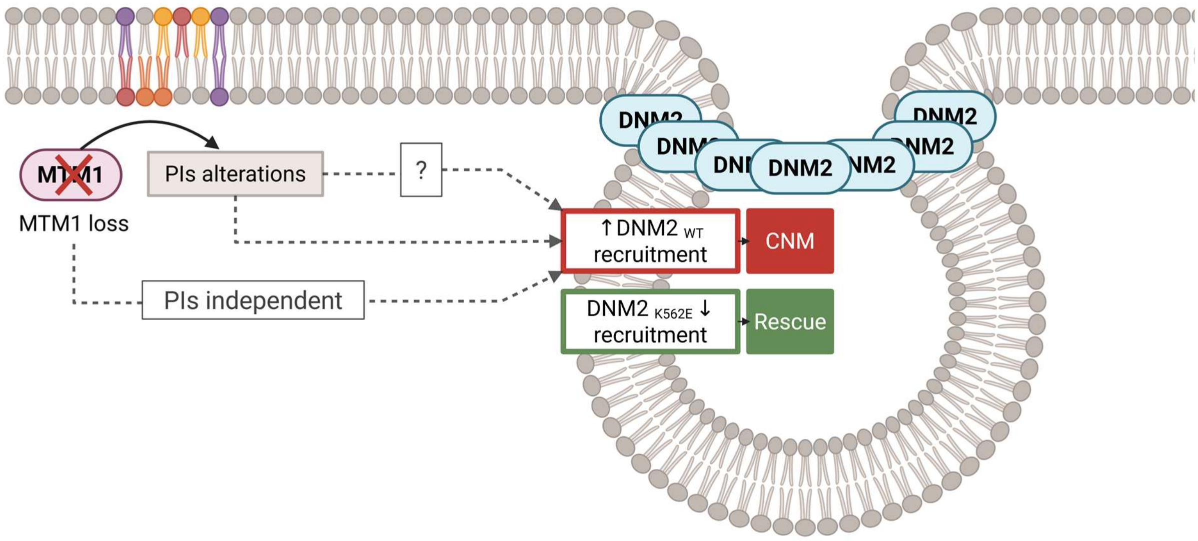


**Supplementary Fig. 6. Hypothetical model linking MTM1, phosphoinositides, DNM2, and CNM pathology and the proposed rescue by the K562E variant.** Schematic representation of a conceptual model illustrating how loss of MTM1 function may alter phosphoinositides (PIs) homeostasis and consequently affect DNM2 membrane occupancy, contributing to the development of centronuclear myopathy (CNM) pathology. This model is intended to provide a conceptual framework for the relationship between MTM1, PIs, and DNM2 in CNM and does not represent mechanistic conclusions derived from new experimental data. Created in BioRender. Laporte, J. (2026) https://BioRender.com/atzx61i.

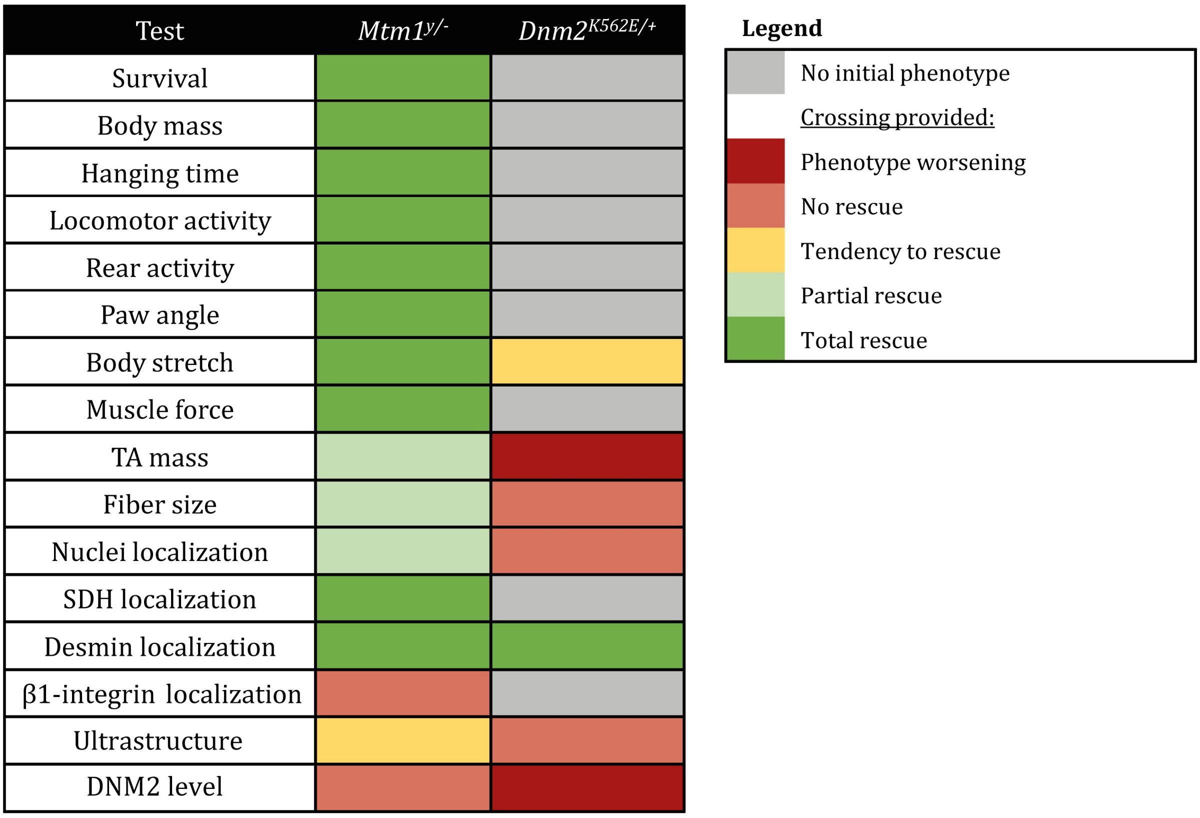


**Supplementary Fig. 7. Overview of the improvements observed when combining the two mutants *Mtm1*$^{-/y}$ and *Dnm2*$^{K562E/+}$ over each individual mouse model.** Summary of whole-body motor performance and muscle phenotypes at 8 weeks, comparing the double mutant to each single mutant. Tendency to rescue= double mutant not statistically different from both single mutant and WT mice. Partial rescue= double mutant differs from both single mutant and WT mice. Rescue= double mutant differs from single mutant but is similar to WT. TA = Tibialis Anterior.

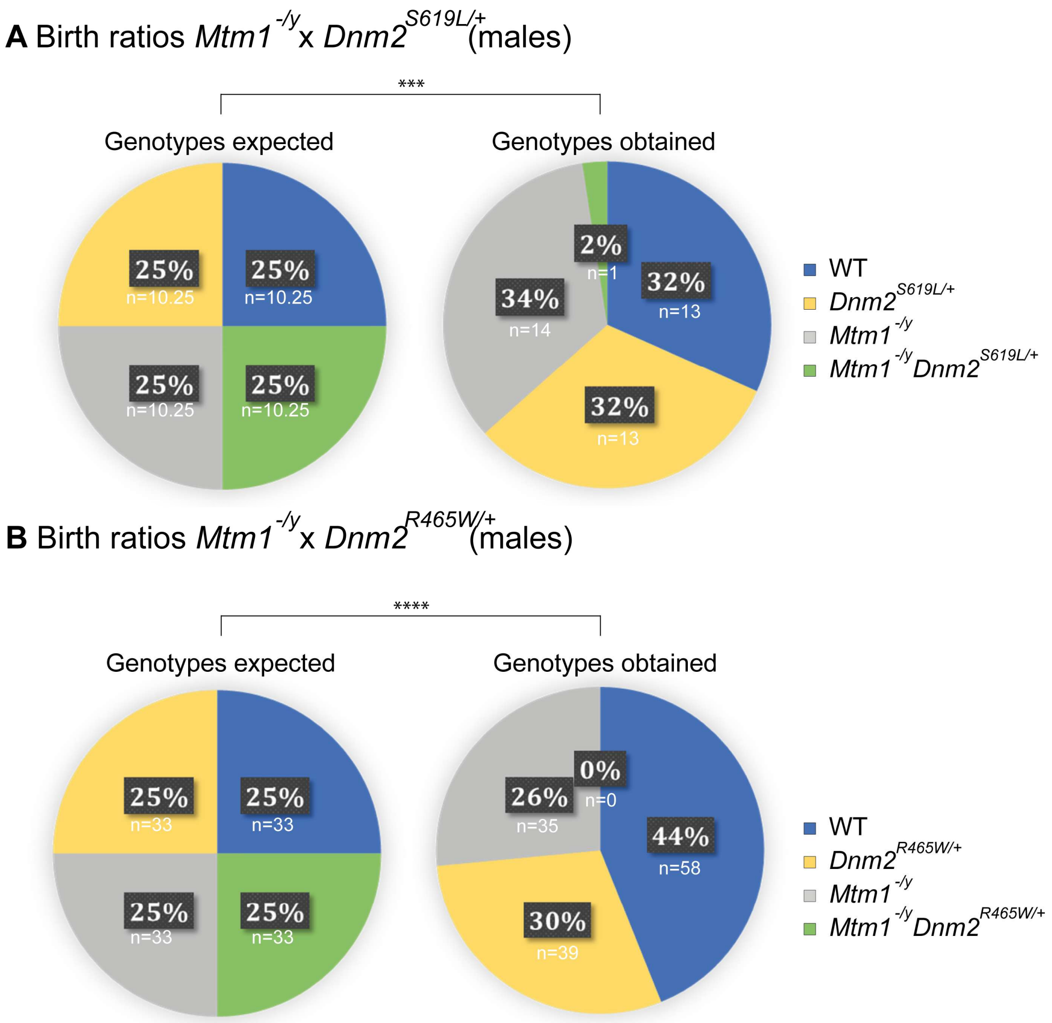


**Supplementary Fig. 8. Perinatal lethality of *Mtm1$^{-/y}$Dnm2$^{S619L/+}$* and *Mtm1$^{-/y}$Dnm2$^{R465W/+}$* mice. (A)** Expected versus obtained birth ratios for male offspring from *Mtm1$^{+/-}$*x *Dnm2$^{S619L/+}$* mice breeding, in percentage and n number (n=41). Only *Mtm1$^{+/-}$* males will develop the myopathy. **(B)** Expected versus obtained birth ratios for male offspring from *Mtm1$^{+/-}$and Dnm2$^{R465W/+}$* crosses, in percentage and n number (n=132). ***p<0.001. ****p<0.0001. (A-B) Chi-squared test.
