# Supplementary material for: DNM2 lipid binding drives centronuclear myopathy and represents a potential therapeutic target
Source: JCI Insight. 2026 May 8;11(9):e204423. doi: 10.1172/jci.insight.204423 (PMC13232018; doi:10.1172/jci.insight.204423)

Western blots
Unedited membranes

Fig. 8E: Western blots DNM2: *Mtm1*$^{-/y}$ mice

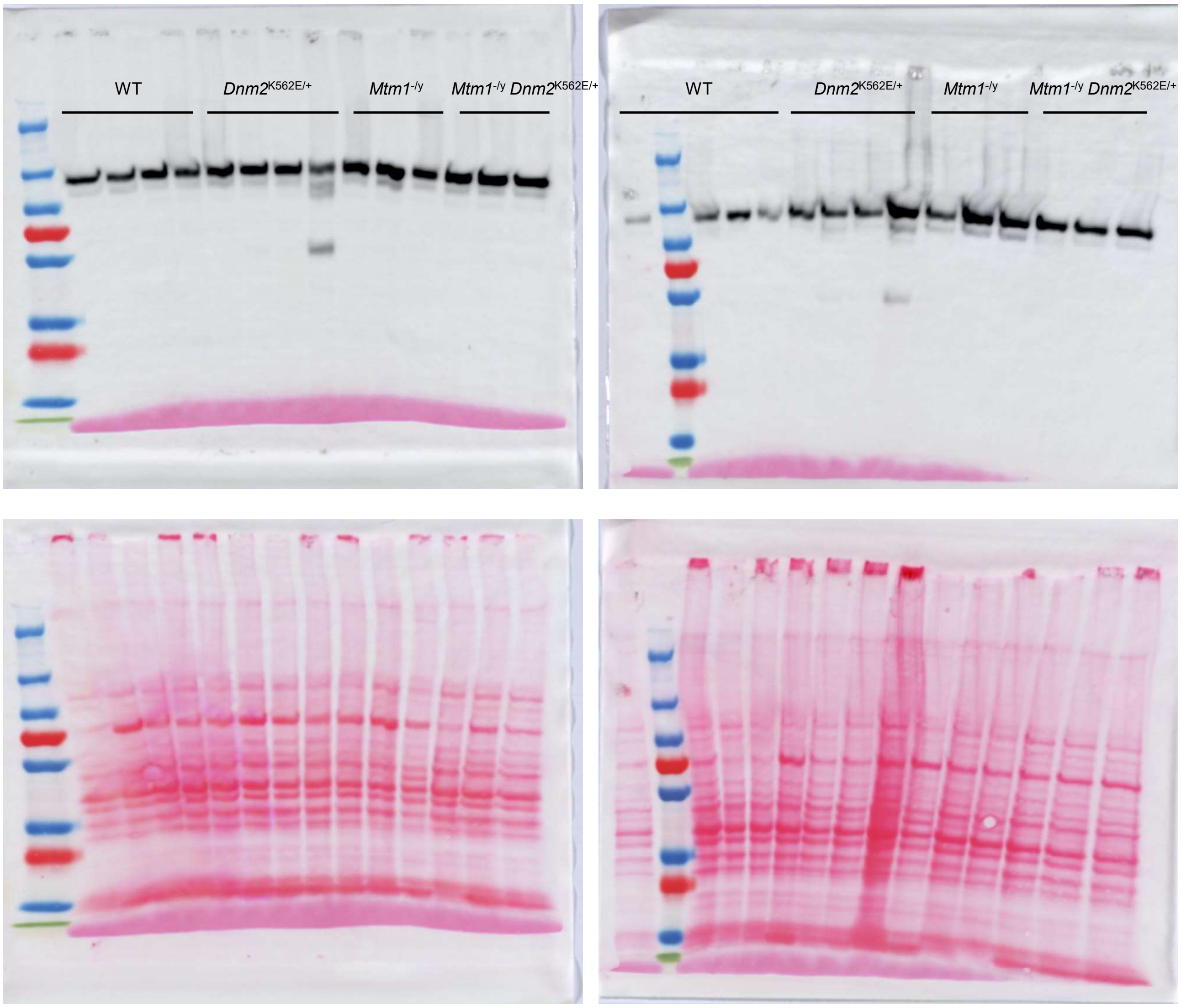

Western blots
Unedited membranes

Supplementary Figure 1B : Western blots DNM2: WT mice

WT–K562E

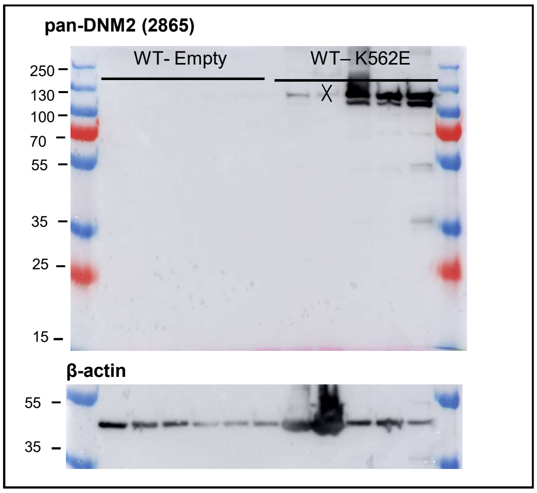

WT–R399A

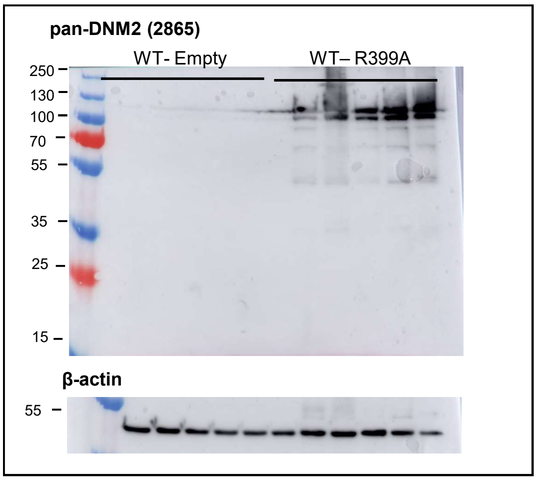

WT-K142A

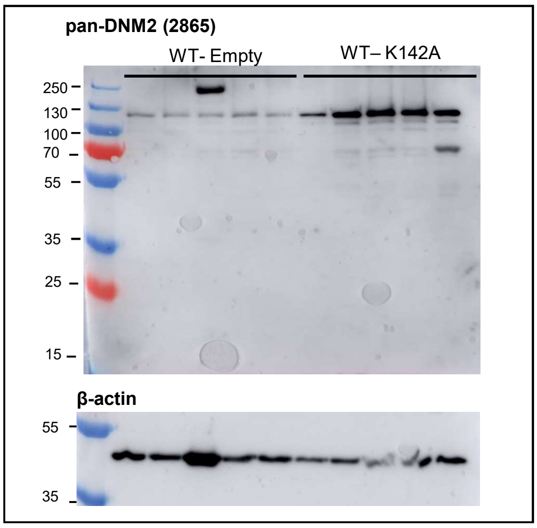

WT–WT DNM2

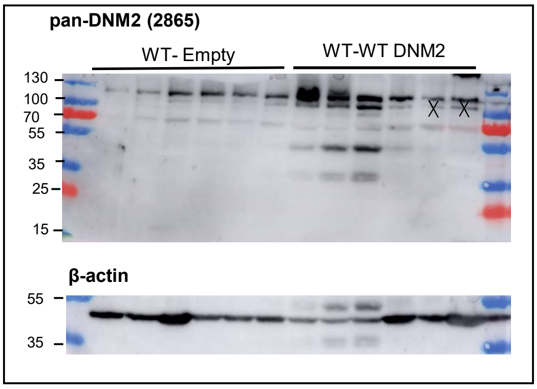

WT–K44A

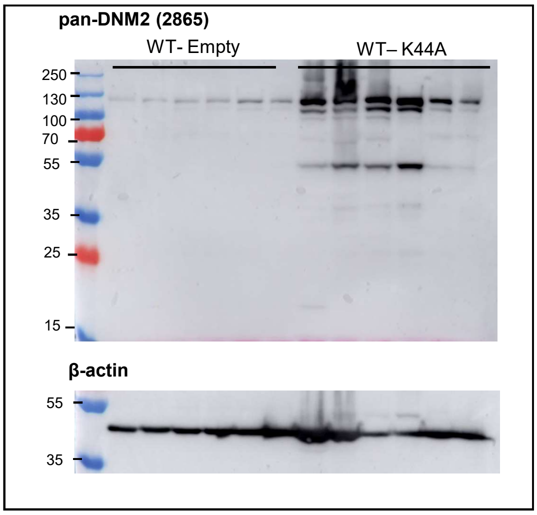

WT–KARW

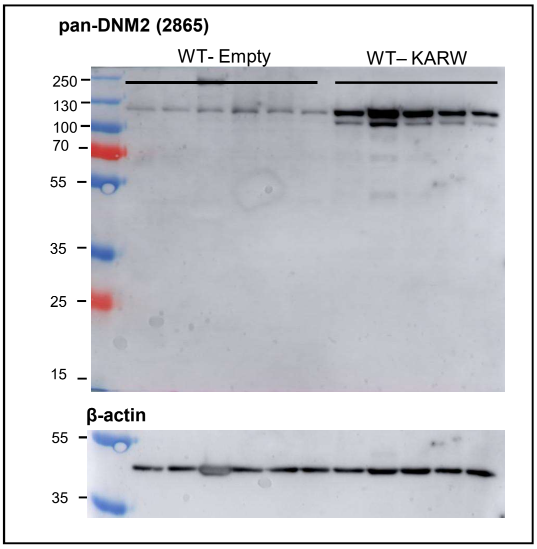

WT-dPRD

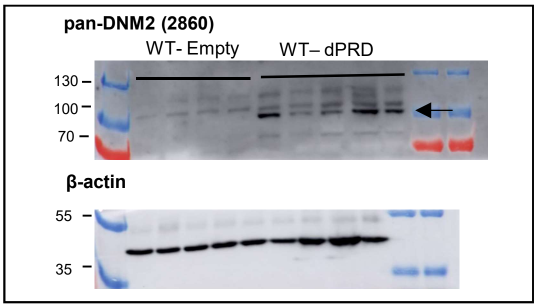

Western blots
Unedited membranes

Supplementary Figure 1F : Western blots DNM2: *Mtm1*$^{y/-}$ mice

Mtm1$^{-/y}$ -K562E

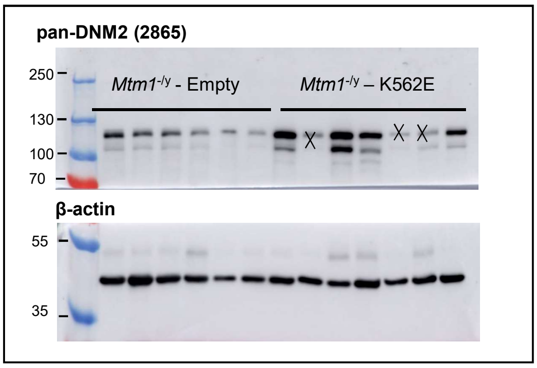

Mtm1$^{-/y}$ –R399A

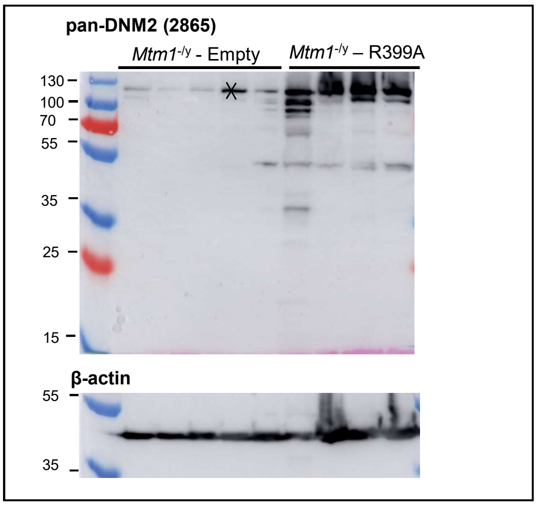

Mtm1$^{-/y}$ –K142A

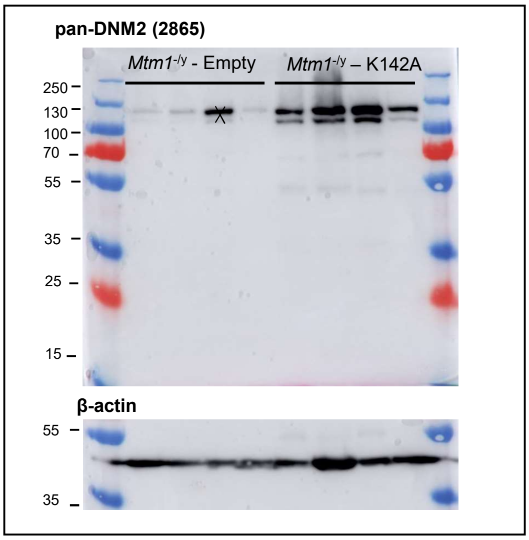

Mtm1$^{-/y}$ –ΔPRD

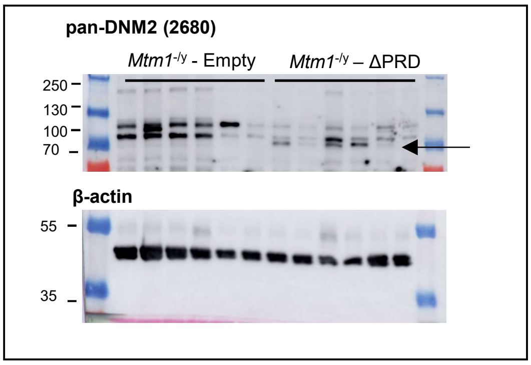

Mtm1$^{-/y}$ –WT DNM2

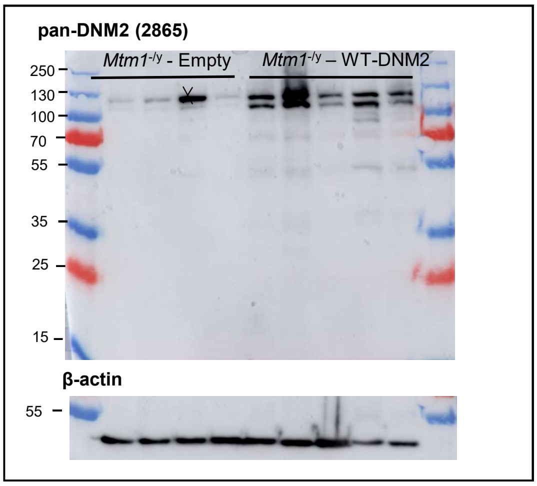

Mtm1$^{-/y}$ –K44A

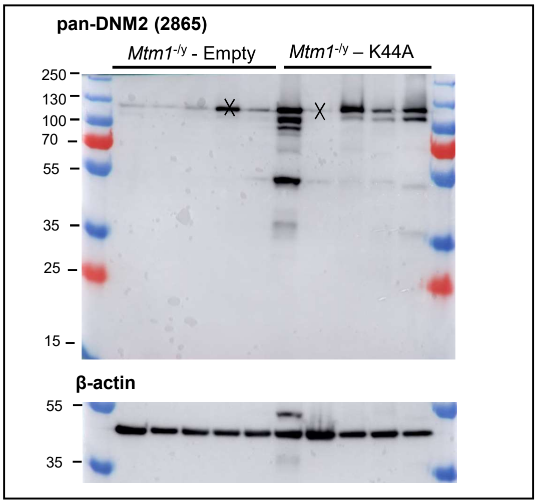

Mtm1$^{-/y}$ –KARW

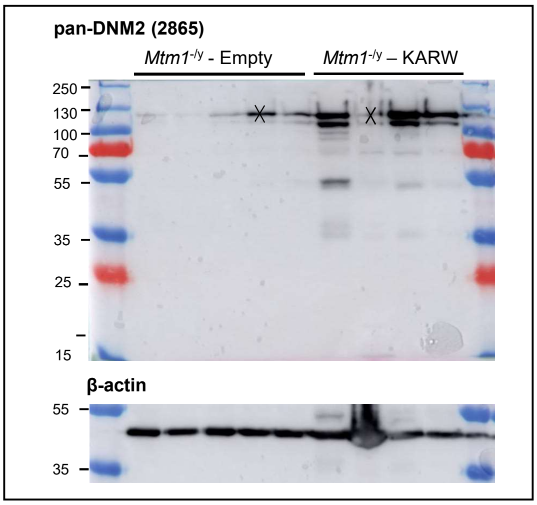

Supplement: Unedited blot and gel images [file jciinsight-11-204423-s208.pdf]
